# Perinatal Brain Injuries in Preterm Infants

**DOI:** 10.3390/cells15201905

**Published:** 2026-10-20

**Authors:** Anet Papazovska Cherepnalkovski, Luka Brajkovic, Klara Cogelja, Lovre Krsan Milostic, Nikolina Vidovic, Angela Renic, Duje Bulat, Jelena Sabljic, Ena Batinovic, Marija Bucat

**Affiliations:** 1 Department of Neonatology, Department for Gynecology and Obstetrics, University Hospital of Split, 21000 Split, Croatia; luka.brajkovic@kbsplit.hr (L.B.); klara.cogelja@kbsplit.hr (K.C.); lovre-krsan.milostic@kbsplit.hr (L.K.M.); nikolina.vidovic@kbsplit.hr (N.V.); angela.renic@kbsplit.hr (A.R.); duje.bulat@kbsplit.hr (D.B.); jelena.sabljic@kbsplit.hr (J.S.); ena.sardelic@kbsplit.hr (E.B.); marija.bucat@gmail.com (M.B.); 2 Faculty of Health Sciences, University of Split, 21000 Split, Croatia; 3 School of Medicine, University of Split, 21000 Split, Croatia

**Keywords:** white matter injury, periventricular leukomalacia, preterm infants, perinatal stroke, developmental outcomes

## Abstract

**Highlights:**

White matter injury and perinatal stroke remain major causes of long-term neurological morbidity in preterm infants. Diffuse non-cystic white matter injury is now the predominant phenotype and is associated with later cognitive, language, behavioral, and motor impairment. Preterm brain injury reflects multifactorial vulnerability involving hypoxia–ischemia, inflammation, oxidative stress, infection, placental pathology, and disturbed cerebral hemodynamics. Serial cranial ultrasonography, MRI-based risk stratification, neuroprotective perinatal care, and multidisciplinary follow-up are essential for early recognition and outcome improvement.

**What are the main findings?**

- Diffuse non-cystic white matter injury is now the predominant preterm brain injury phenotype and is associated with later cognitive, language, behavioral, and motor impairment.
- Perinatal stroke in preterm infants is most often represented by periventricular hemorrhagic infarction, while arterial ischemic stroke and cerebral sinovenous thrombosis are less frequent but clinically important.

**What are the implications of the main findings?**

- Early recognition requires vigilant clinical assessment and systematic neuroimaging, as symptoms of preterm brain injury and stroke may be subtle or nonspecific.
- Neuroprotective perinatal care, MRI-based risk stratification, and multidisciplinary follow-up are essential to improve diagnosis, prognosis, and long-term developmental outcomes.

**Abstract:**

Background: Preterm infants are vulnerable to brain injury because of immature white matter, vascular regulation, and neuroprotective mechanisms. White matter injury (WMI), formerly known as periventricular leukomalacia, is a major cause of cerebral palsy and neurocognitive impairment, while perinatal stroke is an important cerebrovascular cause of adverse outcomes. White matter injury: WMI is linked to hypoxia–ischemia, inflammation or infection, oxidative stress, and impaired oligodendrocyte maturation. It may be cystic or diffuse non-cystic, which is now the most common phenotype. Cystic lesions are strongly associated with motor impairment and spastic cerebral palsy, whereas diffuse injury is increasingly related to cognitive, behavioral, and language deficits. Diagnosis relies on cranial ultrasonography and MRI; prevention focuses on optimized perinatal care, infection control, respiratory stabilization, and neuroprotection. Perinatal stroke: Stroke in preterm infants may occur antenatally or neonatally and includes periventricular hemorrhagic infarction, perinatal arterial ischemic stroke, and cerebral sinovenous thrombosis. Periventricular hemorrhagic infarction is the most common subtype and is often associated with germinal matrix hemorrhage–intraventricular hemorrhage. Risk factors are multifactorial, including fetal, neonatal, placental, thrombotic, infectious, and hemodynamic contributors. Clinical signs may be subtle, making neuroimaging central to classification and prognosis. Conclusion: Despite advances in neonatal care, WMI and perinatal stroke remain major causes of long-term neurological morbidity in preterm infants. Early recognition, systematic neuroimaging, risk stratification, and multidisciplinary follow-up are essential. Future research should identify reliable biomarkers and targeted neuroprotective therapies.

## 1. White Matter Injury in Preterm Infants

### 1.1. Introduction

White matter injury (WMI) in preterm infants, historically described mainly in terms of periventricular leukomalacia (PVL), represents a major cause of cerebral palsy and neurocognitive impairment in prematurely born neonates. While these lesions predominantly affect infants born before 32 weeks of gestation, a comparable pattern of injury occurs in neonates with congenital heart defects, secondary to delayed cerebral maturation induced by in utero hypoxia [1].

In contrast, hypoxic–ischemic encephalopathy (HIE) is defined as an acute encephalopathy triggered by peripartum hypoxia and cerebral ischemia, observed primarily in term neonates. Within the context of HIE, compromised cerebral delivery of oxygen and glucose precipitates cellular energy failure, which correlates significantly with subsequent neurodevelopmental deficits in surviving infants [2].

Fetal development is a highly dynamic process; consequently, gestational-age-dependent maturations within the central nervous system account for the distinct patterns of cerebral injury observed when comparing term and preterm neonates. Whereas term infants with HIE predominantly present with diffuse disease involving the cerebral neocortex, hippocampus, basal ganglia–thalamus, and deep brainstem nuclei, the pathological insult in preterm infants with HIE is characteristically localized within the cerebral white matter [2].

Advances in neuroimaging and epidemiological methodologies have significantly clarified the pathophysiology of preterm WMI. Recent evidence underscores a heritable component, demonstrating that common genetic variants and single-nucleotide polymorphisms (SNPs) may modulate an infant’s susceptibility to, or resilience against, hypoxic–ischemic insult [3].

Genetic factors may modify susceptibility to WMI, and genetic predisposition does not act in isolation. Rather than causing injury directly, common genetic variants lower the threshold at which the developing white matter is damaged by a “multi-hit” cascade of ischemia, excitotoxicity, and oxidative stress. These variants converge on three interdependent biological pathways: the inflammatory cascade, oxidative and excitotoxic stress, and lipid metabolism and structural maturation. Together with neuroepigenetic processes, they strongly influence both the severity of the injury and the capacity of the immature brain to repair itself [4].

Although definitive curative treatments are currently unavailable, contemporary clinical trials are actively investigating preventative strategies and methods to ameliorate long-term neurodevelopmental outcomes [5].

### 1.2. Historical Background and Terminology

The foundational description of neonatal white matter injury was documented by Parrot in 1868, who observed pallid infarctions presenting as yellowish or chalky plaques within the lateral angles of the periventricular white matter [6]. The subsequent liquefaction of these plaques resulted in the formation of cavitary lesions that remained anatomically distinct from the ventricular system. In the early 1960s, Banker and Larroche introduced the term “periventricular leukomalacia” and established its clinico-pathological correlation with hypoxic–ischemic events and spastic cerebral palsy [6]. They characterized neonatal white matter injury as periventricular cystic focal necrosis surrounded by diffuse, non-necrotic damage—the dominant phenotype of that era [6].

With advances in neonatal intensive care, diffuse non-cystic white matter injury has emerged as the predominant phenotype in contemporary cohorts of preterm infants. This condition is radiologically characterized by diffuse non-cystic WMI that might coexist with punctate white matter lesions on early magnetic resonance imaging (MRI), alongside aberrations in white matter microstructure and maturation seen on term-equivalent age MRI scans. To describe the gray matter dysmaturation that concomitantly occurs with diffuse white matter injury, Volpe introduced the term “encephalopathy of prematurity” [7]. These structural modifications underlie the cognitive and behavioral abnormalities observed in modern preterm cohorts, which frequently manifest even in the absence of overt motor deficits [7].

### 1.3. Risk Factors

The risk factors predisposing neonates to preterm white matter injury have been extensively evaluated in prospective cohort studies. Herzog et al. demonstrated that maternal obesity and acute chorioamnionitis elevate periventricular leukomalacia (PVL) risk more than prematurity alone [8]. Similarly, the Italian ACTION study by Gagliardi et al. prospectively evaluated 2085 infants delivered between 23 and 31 gestational weeks (GWs) to determine how early pregnancy disorders impact neonatal outcomes. The investigators categorized maternal conditions into “disorders of placentation” (hypertensive disorders and fetal growth restriction) or “suspected infection/inflammation” (threatened preterm labor and preterm premature rupture of membranes [PPROM]). After adjusting for gestational age, growth-restricted neonates exhibited higher rates of mortality, bronchopulmonary dysplasia, and retinopathy of prematurity. Conversely, cohorts exposed to infectious or inflammatory processes carried a significantly elevated risk of intraventricular hemorrhage (IVH) and PVL [9].

In a subset of 753 extremely premature infants surviving at least 7 days, cystic PVL occurred in 9.2% of cases, peaking at 16% in the 28th gestational week [10]. Prenatal inflammation correlated strongly with PVL pathogenesis. This risk escalated to 22% when intrauterine infection was compounded by PPROM. In contrast, chronic fetal distress, including fetal growth restriction and preeclampsia, showed a minimal association with PVL, carrying a risk under 2% [10]. These data underscore the profound negative impact of PPROM, prolonged rupture of membranes, and prenatal infection on preterm neurodevelopment [2,10].

Perinatal white matter injury in preterm infants is driven by specific prenatal, postnatal, and respiratory risk factors. Prenatally, maternal cocaine abuse, multiple gestation, and placental complications like hemorrhages or vascular anastomoses elevate vulnerability. Postnatally, the risk increases with concurrent intraventricular hemorrhage and conditions causing systemic hypotension and acidosis, including septic shock, necrotizing enterocolitis, and volvulus. Additionally, preterm infants requiring mechanical ventilation face higher risks if they experience episodes of hypocapnia [8,10].

### 1.4. Clinical Manifestations

Advances in neonatal intensive care have reduced mortality among preterm infants; however, this population remains at substantial risk of developmental morbidity in early childhood [11]. White matter injury is a major contributor to long-term motor, cognitive, and behavioral impairment, with neonates born before 32 weeks of gestation consistently showing the highest susceptibility to WMI [5].

### 1.5. Clinical Evolution and Subtypes of White Matter Injury

White matter injury (WMI) is often clinically silent during infancy, becoming more apparent as neurological development advances. Its presentation varies according to the underlying structural subtype: macroscopic cystic WMI, microscopic cystic WMI, or diffuse non-cystic WMI [5].

1. Macroscopic Cystic WMI

Macroscopic cystic WMI, formerly called cystic periventricular leukomalacia, consists of focal necrotic lesions larger than 1 mm. These lesions affect glia, axons, and neural progenitor cells and are usually bilateral in the deep periventricular white matter [5].

Clinically, it often presents in early childhood as spastic diplegic cerebral palsy, mainly affecting the lower limbs. This pattern reflects interruption of the medially positioned corticospinal motor fibers by periventricular cysts. More extensive injury may involve the upper limbs or optic radiation, causing visual deficits [12].

2. Microscopic Cystic WMI

Microscopic cystic WMI consists of small necrotic foci (microscopic necrosis). These lesions are difficult to detect with standard imaging, and their specific neurodevelopmental impact remains poorly defined.

3. Diffuse Non-Cystic WMI

Diffuse non-cystic WMI is now the most common form in preterm infants. MRI typically shows scattered punctate T1 hyperintensities in the periventricular white matter on early scans (PVWMLs, punctate white matter lesions) that might coexist with diffuse non-cystic WMI [13], while advanced imaging may reveal abnormalities in white matter structure, maturation, metabolism, and connectivity [14]. Although often silent in the neonatal period, it increases the risk of later motor, cognitive, socialization, and language difficulties.

### 1.6. Etiology and Pathogenesis

WMI pathogenesis reflects the selective vulnerability of immature white matter, particularly pre-oligodendrocytes, to hypoxic–ischemic injury and related clinical stressors. Cystic WMI results from focal necrosis with loss of all cellular elements after severe hypoxic–ischemic insults, whereas diffuse WMI primarily affects pre-oligodendrocytes and disrupts myelination without prominent frank necrosis [5,12].

Myelination begins in the second trimester and continues into adulthood, progressing caudal-to-rostral from the brainstem to the forebrain, with its most rapid phase during the first two years of life. Oligodendrocytes mediate this process through four developmental stages, from primitive precursors to mature myelin-producing cells [5,15].

Pre-oligodendrocytes predominate between 24 and 30 weeks’ gestation and are highly susceptible to hypoxia–ischemia and inflammation, which trigger oxidative stress and apoptosis [5,16]. Although new pre-oligodendrocytes may proliferate, many undergo maturation arrest and fail to develop into functional myelinating oligodendrocytes [5].

Premature white matter injury is best understood as the product of interacting vascular, inflammatory, glial, oxidative, excitotoxic, and mitochondrial mechanisms that converge on a single, developmentally vulnerable target: the premyelinating oligodendrocyte (pre-OL), the predominant oligodendroglial stage in cerebral white matter during the third-trimester period of peak vulnerability [4,5,12]. Two upstream insults initiate this cascade. Hypoxia–ischemia arises readily because immature white matter contains vascular end and border zones and because cerebrovascular autoregulation is impaired and often pressure-passive. Maternal/fetal infection and systemic inflammation act as a second, potentiating insult, in part because vasoactive cytokines such as TNF-α (tumor necrosis factor-α) and IL-1β (interleukin-1β) further compromise cerebral perfusion. Both insults activate *microglia*, which are concentrated in developing white matter and respond through innate immune receptors such as TLR4 (Toll-like receptor 4), as well as reactive astrocytes; together, these *glial cells* release proinflammatory cytokines (notably interferon-γ and TNF-α), glutamate, and reactive oxygen and nitrogen species (ROS/RNS). Pre-OLs are poorly equipped to withstand this environment. Delayed maturation of their antioxidant enzymes, coupled with the iron they acquire for differentiation, favors hydroxyl radical formation, whereas overexpression of glutamate transporters, Ca^2+^-permeable AMPA (α-amino-3-hydroxy-5-methyl-4-isoxazolepropionic acid)/kainate receptors, and NMDA receptors (N-methyl-D-aspartate receptor) renders them highly susceptible to excitotoxicity. During energy failure, glutamate transporters operate in reverse and raise extracellular glutamate; the resulting Ca^2+^ influx generates further ROS and peroxynitrite, while nitric oxide acts as a mitochondrial poison that triggers apoptosis-inducing factor-mediated, caspase-independent *cell death*. Excitotoxic and free radical injury therefore amplify one another rather than acting in parallel. Importantly, the outcome extends beyond acute pre-OL death and loss of cell processes. Surviving and newly generated oligodendrocyte progenitors frequently remain arrested at the pre-OL stage within a chronically gliotic, inflammatory milieu and fail to differentiate into mature myelinating oligodendrocytes. This persistent maturation arrest, or dysmaturation, accounts for the diffuse hypomyelination, deficient axonal ensheathment, and secondary axonal and neuronal disturbances that characterize the predominantly non-cystic white matter injury observed in contemporary preterm survivors [5,7,12]. This concept is consistent with Volpe’s “encephalopathy of prematurity,” where diffuse WMI may coexist with axonal injury, abnormal neuronal maturation, and reduced gray matter growth [7].

ROS/RNS can also damage both nuclear and mitochondrial DNA, and because impaired DNA repair, particularly of the base excision repair pathway, promotes cell death and genomic instability in other models of brain injury, the DNA repair capacity of pre-OLs may plausibly determine whether these cells die, survive in a maturation-arrested state, or differentiate normally. However, direct evidence that DNA repair capacity or repair failure drives pre-OL injury and dysmaturation in preterm WMI remains limited [4].

A clear mechanistic sequence of events leading to diffuse WMI and gray matter dysmaturation is presented in Table 1.

Recurrent hypoxia–ischemia remains central to preterm WMI and acts synergistically with inflammation and infection [4,5,12].

The association between maternal chorioamnionitis and neonatal WMI is well documented but complex, and it has long been debated whether the injury results directly from infection or indirectly from the preterm birth it causes. Current evidence indicates that chorioamnionitis contributes through both pathways (Table 2) [17,18,19,20,21]. In preterm infants, clinical chorioamnionitis has been associated with a threefold higher risk of cystic PVL and a nearly twofold higher risk of cerebral palsy [17]. Histologic chorioamnionitis has been linked to altered white matter microstructure at term-equivalent age, independent of the degree of prematurity, suggesting that diffuse WMI may begin in utero [18]. Mediation analyses from the Cincinnati Infant Neurodevelopment Early Prediction Study (CINEPS) cohort attributed approximately half of the MRI brain abnormalities [20] and three-quarters of the reduction in motor scores [21] to a direct effect of moderate-to-severe histologic chorioamnionitis rather than to earlier birth. Findings are not uniform, however: in a multicenter cohort, histologic chorioamnionitis was not associated with IVH, punctate WMI, or motor outcome after adjustment [19].

### 1.7. Preventive Strategies

Preventing preterm birth remains the most effective strategy for reducing WMI. Other preventive measures can be grouped into antenatal, perinatal, and postnatal interventions; however, the supporting evidence differs, as few have been shown to reduce WMI itself, whereas most improve broader neonatal or neurodevelopmental outcomes.

Antenatal interventions: *Tocolytics*, such as nifedipine, atosiban, or indomethacin, are used solely to delay preterm delivery, typically by 48 h. This interval allows maternal transfer to a specialized perinatal center and the administration of proven neuroprotective and maturational therapies, rather than providing direct fetal neuroprotection. Tocolysis with nifedipine showed no significant efficacy in preventing neonatal WMI [22].

*Antenatal magnesium sulfate* when given with neuroprotective intent during active preterm labor reduces the incidence of cerebral palsy and/or death [23,24]; however, this neuroprotective benefit is defined by clinical outcomes, and a corresponding reduction in imaging-defined WMI has not been consistently demonstrated.

*Antenatal betamethasone* for pregnancies between 24 and 31 weeks’ gestation is recommended primarily for its broader benefits on neonatal survival and morbidity, whereas its specific effect on WMI is less well established. In a Cochrane meta-analysis of 27 randomized trials (11,272 women; 11,925 neonates), antenatal corticosteroids administered before anticipated preterm birth reduced perinatal death (relative risk [RR] 0.85; 95% confidence interval [CI] 0.77–0.93), neonatal death (RR 0.78; 95% CI 0.70–0.87), and respiratory distress syndrome (RR 0.71; 95% CI 0.65–0.78) (high-certainty evidence) and probably reduced intraventricular hemorrhage (IVH) (RR 0.58; 95% CI 0.45–0.75) and childhood developmental delay (RR 0.51; 95% CI 0.27–0.97). Maternal death, chorioamnionitis, and endometritis probably did not differ between groups, and the benefits were consistent across high-, middle-, and low-resource settings, supporting the continued use of a single course [25].

Prompt diagnosis and treatment of *maternal chorioamnionitis* are supported by the association between intrauterine infection or inflammation and WMI [17,18,20,21], although direct evidence that treatment lowers WMI rates is lacking.

Perinatal interventions: *Delayed cord clamping* has been postulated to improve cardiovascular transition and, consequently, cerebral autoregulation. The timing of umbilical cord clamping represents a simple, low-cost intervention with potential relevance to preterm brain injury. In a Cochrane systematic review of 48 randomized controlled trials involving 5721 infants born at 24 to 36^+6^ weeks’ gestation (with outcome data available for 4884 infants from 40 trials), delayed cord clamping (30–180 s, most commonly 30–60 s) was compared with early clamping (<30 s). Delayed clamping probably reduced mortality before discharge (average risk ratio [aRR] 0.73, 95% CI 0.54–0.98; 20 studies, 2680 infants; moderate certainty) and slightly lowered the incidence of intraventricular hemorrhage (IVH) of any grade (aRR 0.83, 95% CI 0.70–0.99; high certainty). However, it had little or no effect on severe IVH (grades 3–4; aRR 0.94, 95% CI 0.63–1.39) or chronic lung disease (aRR 1.04, 95% CI 0.94–1.14), and the available data were insufficient to determine its effect on periventricular leukomalacia (aRR 0.58, 95% CI 0.26–1.30; low certainty). Notably, a single trial suggested that delayed clamping combined with neonatal care provided with the cord intact may reduce death or neurodevelopmental impairment in early childhood (aRR 0.61, 95% CI 0.39–0.96; 218 infants; low certainty), whereas evidence for umbilical cord milking remained inconclusive. Although the overall certainty of evidence was mostly low, the authors concluded that the cord should not be clamped earlier than 30 s after preterm birth, while the optimal duration of delay remains to be established [26]. At least 30–60 s has therefore been recommended in vigorous term and preterm infants, and this recommendation has been endorsed by the relevant expert societies and resuscitation guidelines [27].

Perinatal risk reduction also includes the prevention and treatment of infection, optimized respiratory support, and avoidance of systemic blood pressure instability and postnatal hypotension. These measures target recognized risk factors for WMI, such as infection, hypocapnia, and hypotension; however, their benefit is inferred mainly from these associations rather than from trials demonstrating a reduction in WMI itself.

Postnatal interventions: An 11-year follow-up of the Caffeine for Apnea of Prematurity (CAP) randomized clinical trial showed that neonatal *caffeine citrate therapy* for apnea of prematurity was associated with a significantly lower risk of motor impairment but did not significantly alter the rate of combined functional impairment or general academic and behavioral outcomes [28]. This benefit therefore concerns broader neurodevelopmental outcomes rather than proven prevention of WMI.

*Targeted nutrition* is an integral part of supportive care, but evidence for a specific effect on WMI is limited.

In preterm infants, *low-dose erythropoietin* has been associated with less neurological disability (RR 0.40; 95% CI 0.27–0.59) and better cognitive outcomes [29], and a Cochrane review reported fewer cases of IVH, PVL, necrotizing enterocolitis, and neurodevelopmental impairment (RR 0.62; 95% CI 0.48–0.80), although the evidence was of low quality [30]. However, the two largest high-dose trials, including the Preterm Erythropoietin Neuroprotection Trial (PENUT) of 941 extremely preterm infants (RR 1.03; 95% CI 0.81–1.32), found no reduction in death or neurodevelopmental impairment; therefore, routine use for neuroprotection is not supported [31].

### 1.8. Diagnostic Evaluation

Diagnosis relies on serial cranial ultrasonography (CUS) and MRI. Ultrasonography is reliable for detecting cystic WMI and is preferred for longitudinal neonatal intensive care monitoring because it is bedside-accessible. Serial scanning is essential, as cystic lesions evolve over time and typically become visible 2–6 weeks after injury [32].

**Figure 1:**
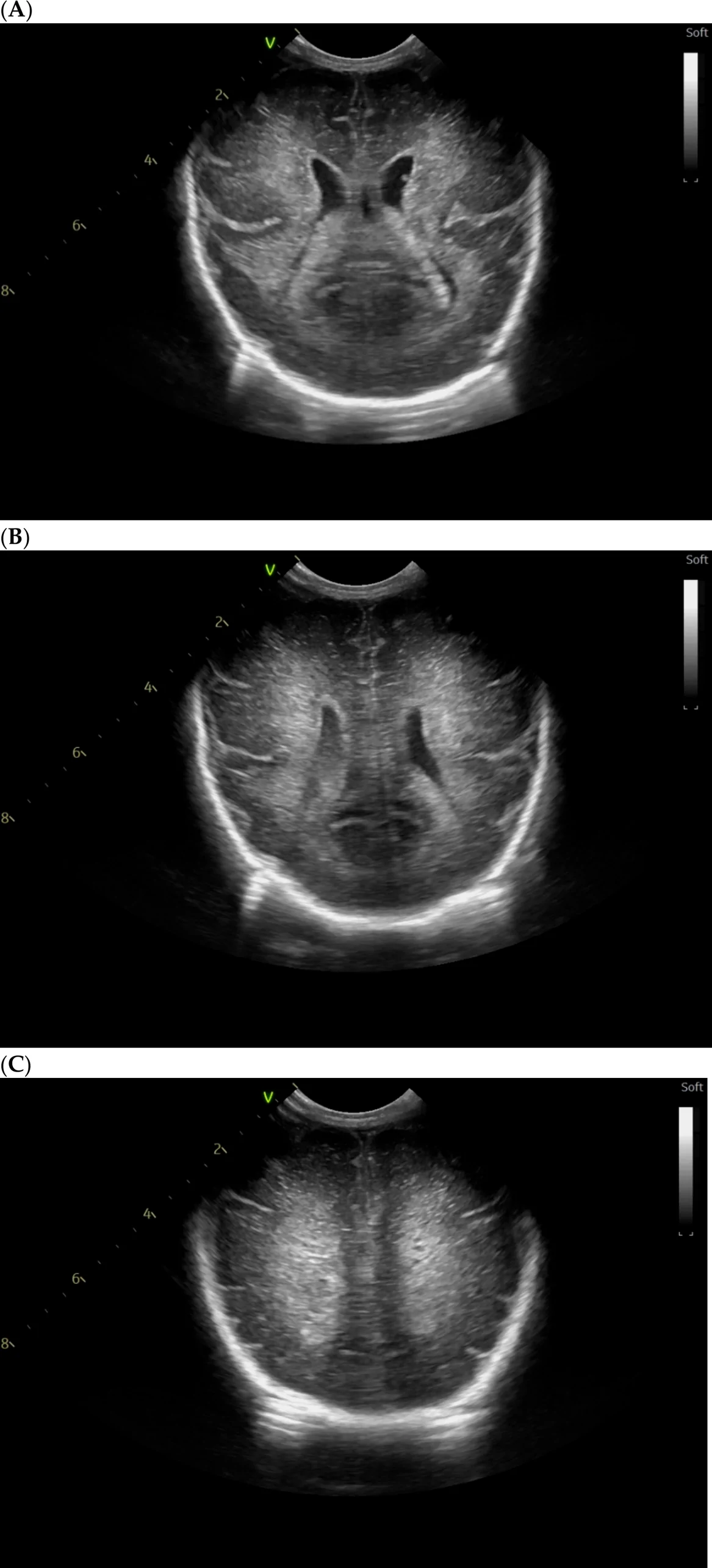

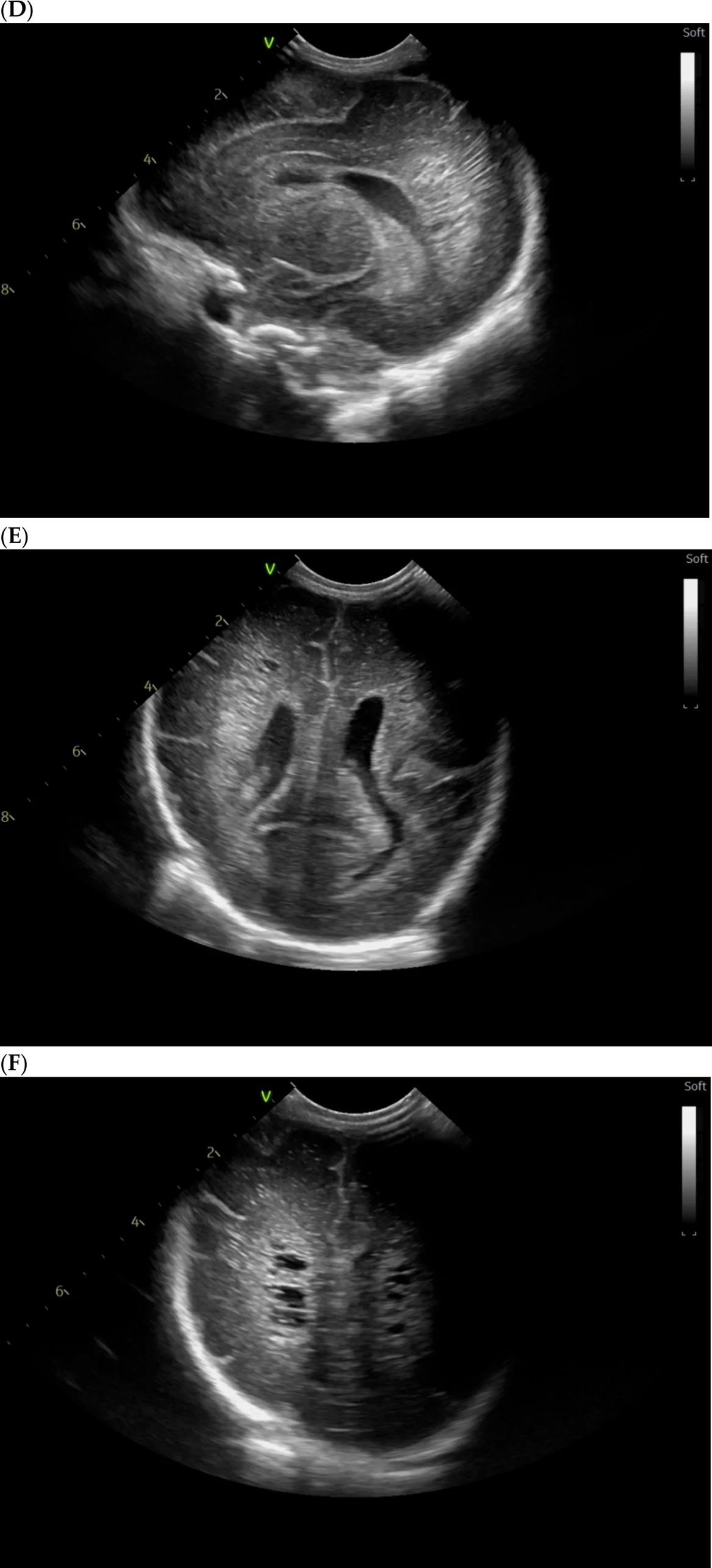

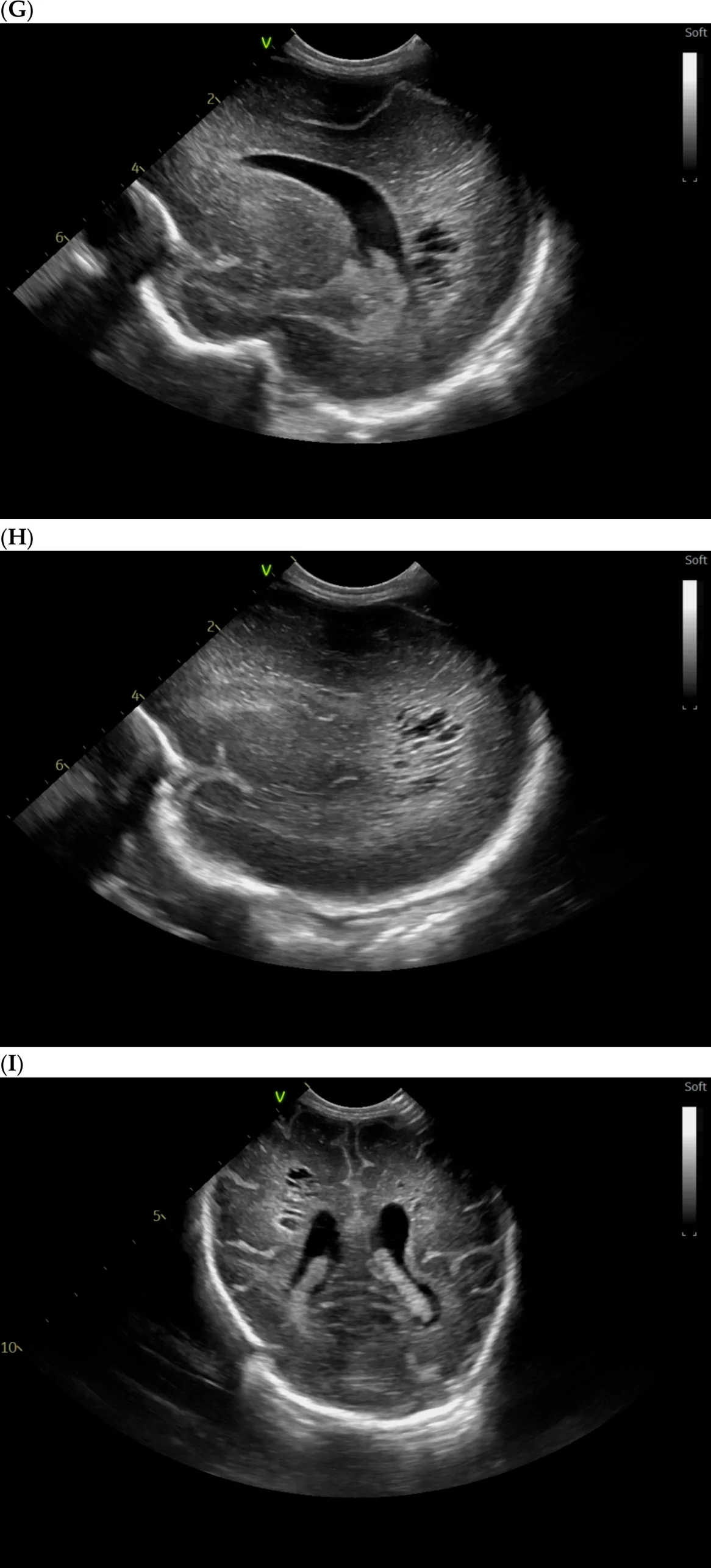

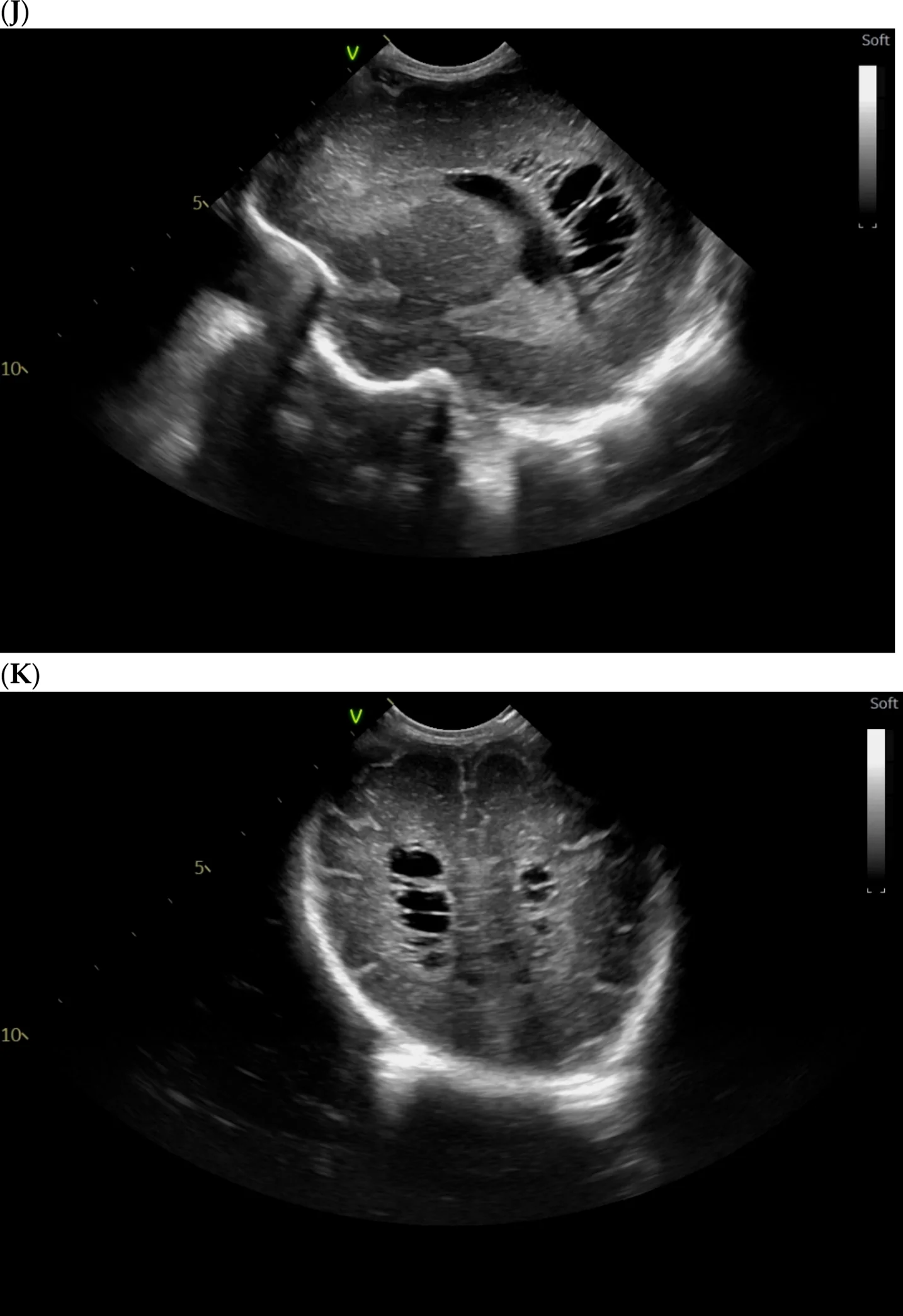
Development of periventricular cystic leukomalacia (grade 3 PVL) in an extremely preterm newborn infant, as demonstrated by cranial ultrasonography. (**A**–**C**) Rarefaction within the periventricular hyperechogenicity on day of life (DOL) 10. (**D**) Clear formation of occipital cysts on DOL 13. (**E**–**H**) Multiple periventricular cysts extending into the deep temporal and occipital periventricular white matter on DOL 16. (**I**–**K**) Confluent cystic lesions in the typical areas on DOL 29.

Cystic spaces may later resolve, leaving ex vacuo ventriculomegaly detectable on term-equivalent ultrasound. The historical ultrasound-based classification of cystic PVL by De Vries et al. includes four stages of injury as follows [33,34]:

1. **Grade I:** Transient periventricular echogenicity persisting for more than 7 days.
2. **Grade II:** Localized cystic lesions confined to the external angles of the lateral ventricles.
3. **Grade III:** Extensive cystic formations localized within the frontoparietal and occipital periventricular white matter (cystic PVL) (Figure 1).
4. **Grade IV:** Extensive cystic lesions extending into the subcortical white matter (cystic subcortical leukomalacia).

This grading system has been used to describe the progression of cystic white matter lesions rather than as a contemporary framework for all forms of WMI, and its limitations are most evident for grade I hyperechogenicity. CUS features of periventricular hyperechogenicity supporting grade I PVL include a patchy appearance, extension beyond the peritrigonal area, and a bilateral but asymmetric periventricular distribution [32]. Dammann and Leviton classified hyperechogenicities by duration as brief (1–6 days), intermediate (7–13 days), or prolonged (≥14 days) [35]; prolonged hyperechogenicities have also been shown to predict white matter abnormalities on MRI [36]. Some authors propose the term “persistent echogenicities” to better describe the mild form of WMI (formerly grade I) [32].

Although cranial ultrasonography has limited sensitivity for diffuse non-cystic WMI, advances in ultrasound equipment and scanning techniques now allow serial scans to adequately characterize diffuse WMI on later examinations. WMI frequently results in subtle ventriculomegaly. On late scans, impaired head growth is often accompanied by enlarged extracerebral spaces, a widened interhemispheric fissure, and mild ex vacuo ventriculomegaly with irregular ventricular borders, findings suggestive of atrophy [37]. Reduced complexity of post-primary gyration may be accompanied by sulci extending almost to the lateral ventricles. Reflecting diffuse WMI, these features become apparent only on late scans, near or at term-equivalent age, and are not included in the De Vries classification [32].

Figure 1 shows the evolution of bilateral cystic PVL on cranial ultrasonography in an extremely preterm infant born at 27 + 3 GW and followed until discharge from the maternity ward. Pronounced periventricular white matter hyperechogenicity was visible immediately after birth. On day of life (DOL) 10, rarefaction and small occipital cysts developed within these hyperechoic zones, as seen in the 4th–6th coronal planes (panels A–C). On DOL 13, occipital cysts were clearly formed in the left parasagittal plane (panel D). On DOL 16, multiple periventricular cysts extended into the deep temporal and occipital periventricular white matter in both the coronal and parasagittal planes (panels E–H). Subsequently, the cystic lesions in these areas became more extensive and confluent, consistent with the “Swiss-cheese” appearance (panels I–K).

CUS and MRI provide complementary information in the assessment of WMI of prematurity. CUS is well suited to sick or very preterm infants and reliably identifies the major lesions that predict adverse neurodevelopmental outcome, namely germinal matrix–intraventricular hemorrhage (GMH-IVH), hemorrhagic parenchymal infarction, and cystic WMI, but it has limited sensitivity for focal, punctate, or diffuse WMI. MRI, the current reference neuroimaging modality, more accurately characterizes the nature and extent of white matter abnormalities, including diffuse WMI and punctate lesions, and enables assessment of myelination of the posterior limb of the internal capsule at term-equivalent age (TEA), a predictor of motor outcome [32]. Accordingly, some neonatal units now perform routine MRI in very preterm infants, either as a single scan before discharge or at TEA, or as two scans: an early scan at approximately 32 weeks’ postmenstrual age to detect acute punctate lesions, followed by a second scan at TEA. Because the appearance of WMI evolves over time, as best documented by serial CUS, MRI alone may underestimate its severity, particularly in limited cystic PVL; the two modalities should therefore be combined [13,32].

Various MRI-based systems have been developed for grading WMI. In the scheme of Miller et al., grading relies predominantly on focal changes in T1-weighted images, specifically in how many punctate hyperintense lesions are present and whether hypointense regions suggestive of cysts can be seen [13,38]. Four categories of severity are distinguished: normal, with no white matter lesions; minimal, with three or fewer foci of abnormal T1 signal, each smaller than 2 mm; moderate, with more than three such foci or foci larger than 2 mm, but with less than 5% of the hemisphere affected; and severe, with more than 5% of the hemisphere affected. The score has proved reliable between raters and useful for predicting outcome at follow-up [38,39]. Although both approaches share similar elements, early MRI scoring focuses primarily on characterizing WMI lesions, whereas TEA scores, as proposed by Kidokoro et al. [40], capture WMI sequelae such as cystic lesions, signal abnormalities, delayed myelination, and white matter volume loss. Roychaudhuri et al. demonstrated a strong association between the systematic assessments of WMI at the two time points (*p* = 0.007) and global injury severity (*p* < 0.001) [39]. Furthermore, Maalouf et al. observed diffuse and excessive high signal intensity (DEHSI) in the white matter on T2-weighted images at term in most infants born at a gestational age (GA) below 30 weeks; because DEHSI was commonly associated with imaging signs suggestive of cerebral atrophy, including ventricular dilation and widening of the extracerebral space, they proposed it as a marker of white matter disease [36]. A very recent systematic review explored the combined effect of electroencephalography (EEG) and MRI for improved prediction of neurodevelopmental outcome in preterm infants. In this review of 12 studies (1630 infants born at 22 to 36^+6^ weeks’ gestation), pooled data from 952 infants showed that EEG alone predicted abnormal neurodevelopment with a sensitivity of 64% (95% CI 48–80%) and a specificity of 70% (95% CI 59–81%). MRI alone had the same sensitivity (64%, 95% CI 48–80%) but a higher specificity (89%, 95% CI 81–97%). When EEG and MRI were combined (3 studies, 372 infants), sensitivity rose to 70% (95% CI 49–90%) and specificity to 96% (95% CI 93–100%), outperforming either test alone. However, the evidence remains limited, as multimodal neurological assessment is still an emerging practice in neonatal care [41].

### 1.9. Therapeutic Interventions

Although the temporal evolution and long-term consequences of cystic WMI are well recognized, no specific therapy is currently available. Perinatal WMI evolves through three phases. In the *acute phase*, initial hypoxia–ischemia, excitotoxicity, and systemic inflammation cause focal necrotic cell death in the periventricular regions. The *secondary phase*, occurring hours to days later, is characterized by mitochondrial dysfunction, free radical production, and secondary energy failure. The *tertiary phase*, which may evolve over weeks to months, is defined by chronic neuroinflammation, astrogliosis, and failure of pre-OLs to mature into myelinating oligodendrocytes, leading to permanent hypomyelination [5,12]. Current research is investigating several therapeutic modalities targeting these distinct phases: therapeutic hypothermia, stem cell transplantation, recombinant erythropoietin administration, and anti-inflammatory approaches (Table 3).

1. Therapeutic hypothermia (TH)

Hypothermia was considered a modality that might potentiate common problems of prematurity, such as hypotension, brain hemorrhage, and breathing difficulties [42]. So far, the clinical evidence is mixed. One study compared 36 newborns who were cooled outside the standard criteria (e.g., infants born at 34–35 weeks, infants who collapsed after birth, or infants with heart disease) with 129 infants cooled according to protocol. Both groups had similar complication rates and similar neurological outcomes at 18–20 months. However, five of the infants cooled outside the criteria developed intracranial hemorrhage and had poor outcomes [43]. A retrospective study gave a less encouraging picture. It compared 31 cooled infants born at 34–35 weeks with 32 cooled term infants. Preterm infants had higher mortality (12.9% vs. 0%, *p* = 0.04) and more white matter damage (66.7% vs. 25.0%, *p* = 0.001) [44].

Animal studies show that cooling can protect the preterm brain, but only under certain conditions. In fetal sheep at a stage of development like the human preterm infant, brain cooling for 72 h (extradural temperature 29.5 ± 2.6 °C) started 90 min after severe asphyxia. It protected the basal ganglia and hippocampus, preserved immature oligodendrocytes in the periventricular and parasagittal white matter, and reduced both microgliosis and apoptosis [45,46].

TH has also been tested in a prospective trial. In a multicenter randomized controlled trial of infants with HIE born at 33–35 weeks, starting hypothermia within 6 h of birth did not reduce death or disability at 18–22 months’ corrected age [47]. Cooling is, however, less likely to be used in extremely preterm infants. These infants often develop several complications (IVH, necrotizing enterocolitis [NEC], late-onset sepsis, bronchopulmonary dysplasia [BPD], and apnea), which increase their risk of death or disability by 2 years of age. In contrast, moderate and late preterm infants’ brain injury more often follows a clear event at a known time that could allow a similar approach to that used for infants born at ≥36 weeks’ gestation.

2. Recombinant erythropoietin

Although showing promising results in preclinical trials, routine use for preterm neuroprotection is not supported according to the results of the large clinical trials discussed previously [29,30,31].

3. Stem cell transplantation

Cellular therapies—particularly using mesenchymal stem cells (MSCs) or umbilical cord blood cells—aim to alter the microenvironment of the tertiary phase. They function primarily through paracrine signaling, releasing trophic factors and extracellular vesicles (exosomes) that cross the blood–brain barrier. Preclinical models show that MSCs can stimulate pre-OL survival and force them past their maturation arrest to promote true remyelination. Clinical trials (such as phase 1/2 safety trials combining MSCs with hypothermia) have shown excellent safety and feasibility profiles, though large-scale efficacy data are still being gathered and clinical evidence in preterm WMI remains limited [48].

4. Anti-inflammatory approaches

Because chronic neuroinflammation drives pre-OL maturation arrest in the tertiary phase, researchers are exploiting the prolonged “therapeutic window” of this phase. Current investigation centers on stem-cell-derived exosomes (MSC-Exos). Preclinical research demonstrates that these cell-free therapies successfully block inflammatory cascades, mitigating the chronic inflammatory drive that sustains cystic progression [49].

**Table 3:** Summary of comparative progress.

Therapeutic Strategy	Target Phase of Evolution	Current Research Status	Major Hurdle/Limitation
Therapeutic Hypothermia	Acute/Secondary	Standard for term HIE; experimental for preterm WMI	High complication/mortality rate in preterm infants
Stem Cell Therapy	Secondary/Tertiary	Transitioning from preclinical to Phase I/II trials	Scalability, standardized dosing, and cell survival
Recombinant EPO	Acute through Tertiary	Extensively trialed; negative Phase III clinical results	Failed to show long-term cognitive/motor benefit in large trials
Anti-Inflammatory	Tertiary (Weeks/Months)	Highly active preclinical focus (e.g., exosomes)	Standardizing isolation protocols and clinical entry

Abbreviations: EPO, erythropoietin; HIE, hypoxic–ischemic encephalopathy; WMI, white matter injury.

### 1.10. Conclusions

Despite major advances in neonatal intensive care, WMI remains a leading cause of long-term neurological morbidity in preterm infants. Although cystic PVL has become less frequent, the overall burden of WMI has not declined substantially, as diffuse non-cystic injury, often clinically silent in the neonatal period, now predominates and underlies later motor, cognitive, behavioral, and language impairment. Because injury frequently originates in utero from the interplay of hypoxia–ischemia, intrauterine infection, and inflammation, and then evolves through acute, secondary, and tertiary phases that converge on pre-OL maturation arrest, effective neuroprotection must span the entire perinatal continuum rather than rely on postnatal care alone.

*Prenatally*, priorities include prevention of preterm birth, rigorous surveillance of high-risk pregnancies, prompt recognition and treatment of chorioamnionitis, and timely administration of antenatal corticosteroids and magnesium sulfate, supported where appropriate by short-term tocolysis to allow maternal transfer to a perinatal center. *Perinatally*, delayed cord clamping, optimized respiratory support with avoidance of hypocapnia, infection control, and hemodynamic stability should be combined to limit secondary injury. *Postnatally*, serial cranial ultrasonography complemented by MRI at term-equivalent age enables early detection and risk stratification, while caffeine therapy, targeted nutrition, and structured multidisciplinary neurodevelopmental follow-up support optimal outcomes. However, most of these interventions improve broader neonatal or neurodevelopmental outcomes rather than demonstrably reducing WMI itself, and no therapy specifically targeting established injury is yet available. Reducing the burden of WMI will therefore require validated biomarkers of fetal and neonatal brain injury, standardized imaging-based outcome measures, and well-designed trials of phase-specific neuroprotective and regenerative strategies, particularly those targeting the prolonged tertiary window.

## 2. Perinatal Stroke in Preterm Infants

### 2.1. Introduction

Perinatal stroke is defined as a vascular injury of the developing brain occurring between the 20th week of gestation (GW) and the 28th postnatal day [50]. It is mostly diagnosed in neonates presenting with acute neurological symptoms; however, in some cases, the diagnosis is established later in childhood, when the first clinical manifestations emerge, most frequently as hemiparesis. Perinatal stroke should therefore be considered in any newborn with unexplained neurological signs, and neuroimaging is essential to characterize the infarct subtype and guide subsequent management.

Classification of perinatal stroke is based on clinical and neuroimaging characteristics, including the timing of symptom onset, the vascular territory involved, and the ischemic or hemorrhagic nature of the lesion. Advances in neuroimaging have enabled the delineation of six distinct disease entities in term neonates, thereby supporting more precise research stratification and therapeutic decision-making: 1. neonatal arterial ischemic stroke (AIS), 2. neonatal hemorrhagic stroke (HS), 3. cerebral sinovenous thrombosis (CSVT), 4. arterial presumed perinatal ischemic stroke (APPIS), 5. presumed perinatal hemorrhagic stroke (PPHS), and 6. in utero periventricular venous infarction (PVin) [51,52]. The first three entities represent acute neonatal stroke syndromes, with symptom onset and diagnosis occurring during the neonatal period, whereas the latter three are frequently identified later, when asymmetric motor development raises suspicion for hemiparetic cerebral palsy.

Although the underlying pathophysiological mechanisms often remain incompletely defined, targeted treatment strategies and neuroprotective interventions may contribute to improved outcomes.

Most survivors experience long-term sequelae, most notably cerebral palsy, as well as epilepsy, learning difficulties, and behavioral challenges, all of which may substantially affect children and their families [52]. Nevertheless, the plasticity of the developing brain may facilitate functional recovery, and outcomes can be more favorable than those observed following comparable lesions in adults.

Stroke may also occur in preterm infants, defined as those born before 37 completed weeks of gestation, either in utero or during the postnatal period. Most studies of perinatal stroke exclude preterm infants, whereas investigations of preterm brain injury have traditionally focused on white matter injury or germinal matrix hemorrhage–intraventricular hemorrhage (GMH-IVH). A major complication associated with GMH-IVH is periventricular hemorrhagic infarction (PVHI), which is classified as a venous stroke [53]. According to a recent systematic review, PVHI accounted for 80% of stroke cases in preterm infants, whereas 17% were classified as perinatal arterial ischemic stroke (PAIS) and 3% as CSVT [54]. Another study reported a higher-than-expected incidence of PAIS in preterm infants [55].

**Periventricular hemorrhagic infarction (PVHI):** PVHI is characterized by hemorrhage and associated parenchymal injury located adjacent to the cerebral ventricles. Periventricular hemorrhagic infarction, previously classified as grade IV intraventricular hemorrhage (IVH), results from infarction secondary to impaired venous drainage of the medullary veins within the white matter following germinal matrix hemorrhage–intraventricular hemorrhage (GMH-IVH) and is pathophysiologically distinct from cerebral sinovenous thrombosis (CSVT) [12]. This circulatory disturbance occurs in the subependymal region, where the medullary veins drain into the terminal vein [12,56,57]. Parietal PVHI has been reported more frequently than temporal or frontal-lobe PVHI [57,58], and bilateral involvement occurs in 10% to 15% of infants [56].

**Figure 2:**
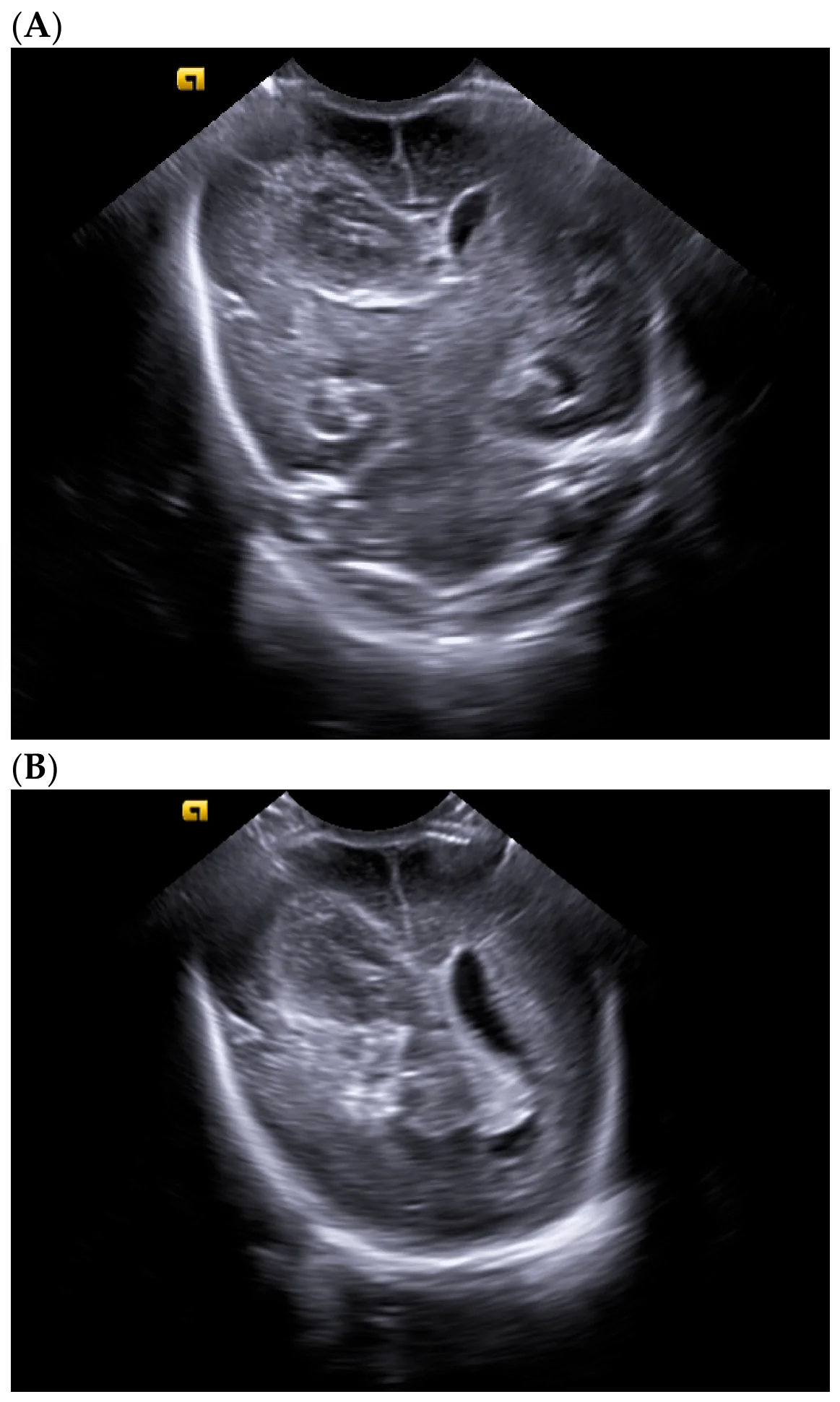

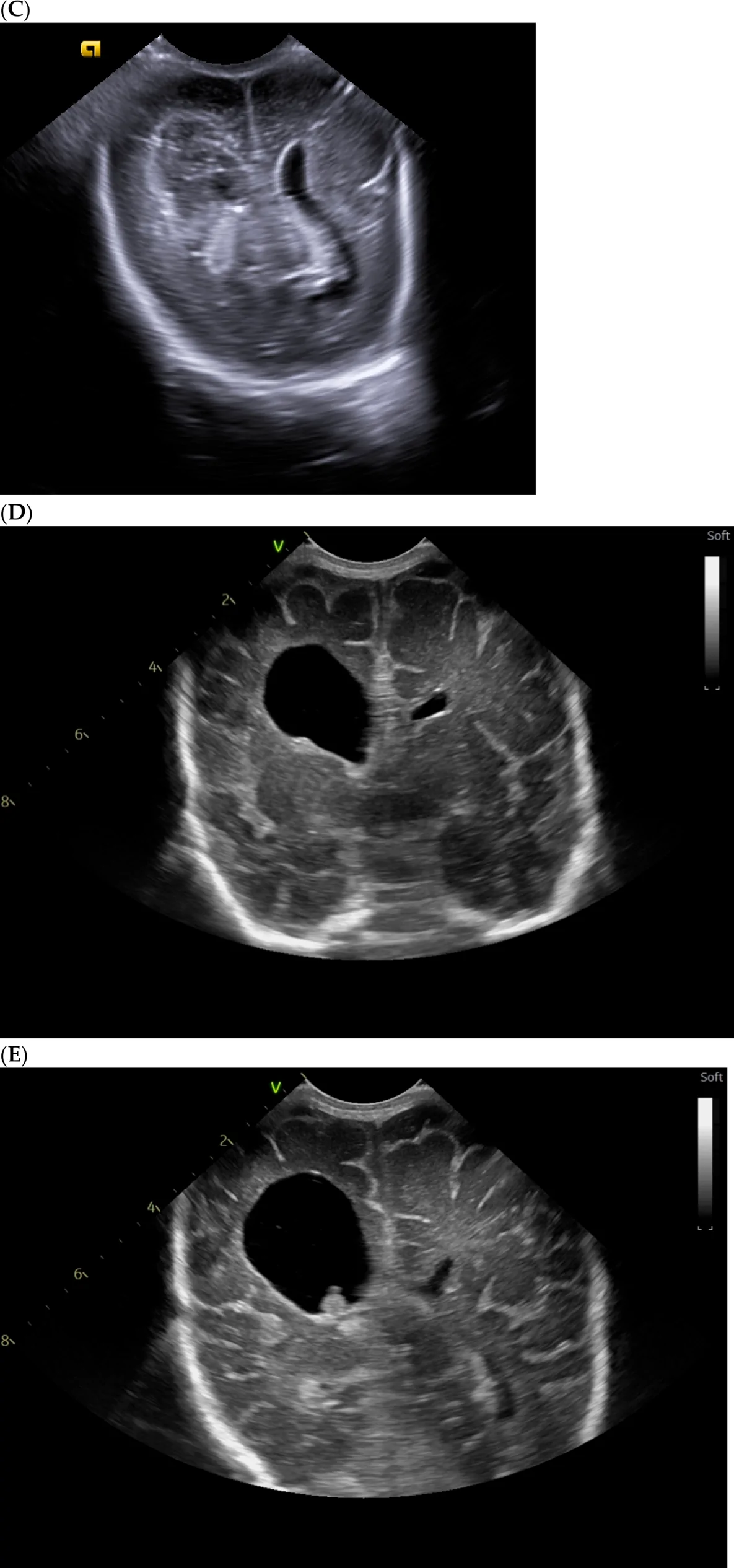

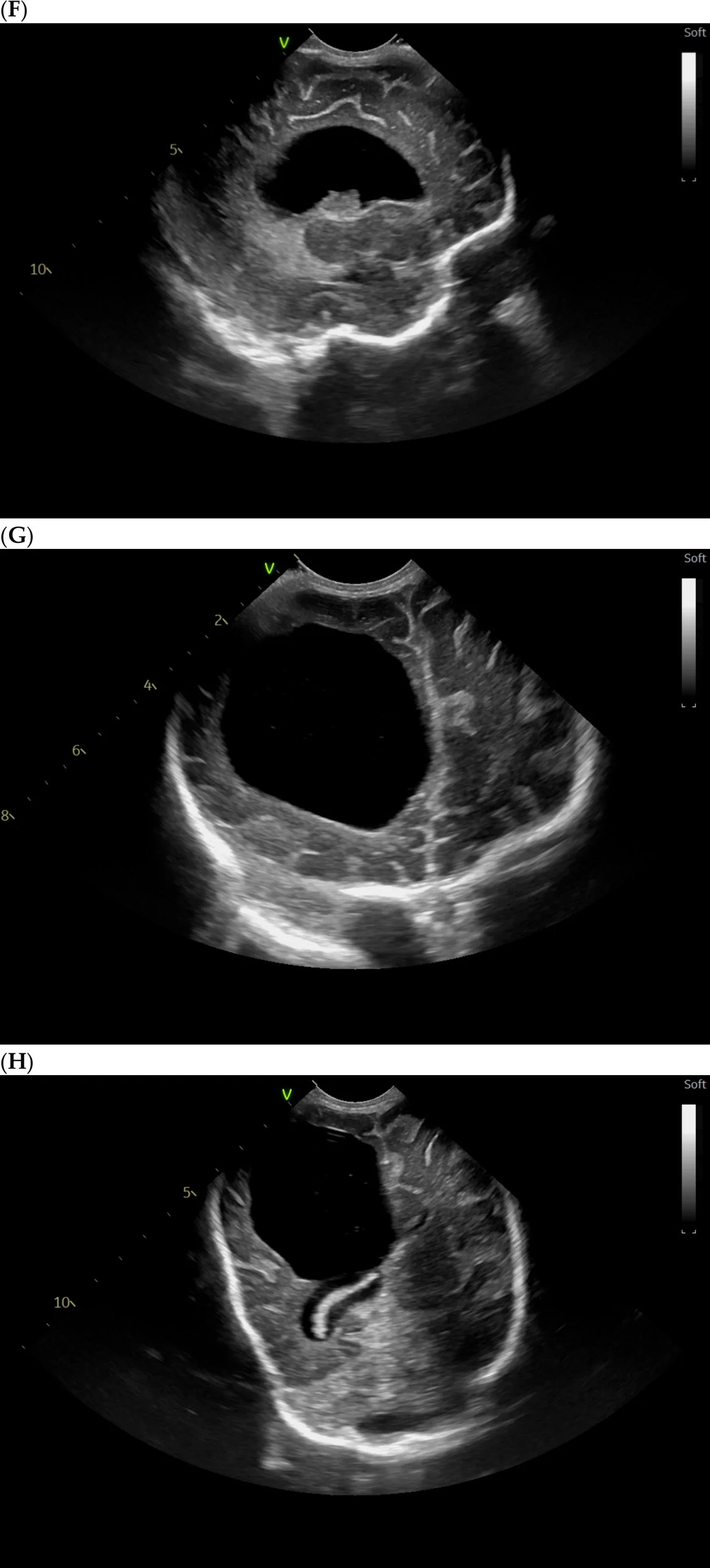
Development of unilateral periventricular hemorrhagic infarction (PVHI) in an extremely preterm newborn infant born at 23 + 2 gestational weeks (GWs), with subsequent unilateral hydrocephalus and ventriculoperitoneal shunt placement at 6 months of age, as demonstrated by cranial ultrasonography. (**A**–**C**) Left-sided germinal matrix hemorrhage–intraventricular hemorrhage (GMH-IVH) with evolving PVHI on day of life (DOL) 14. (**D**–**F**) Left-sided hydrocephalus without signs of intracranial hypertension at 4 months of age. (**G**,**H**) Hypertensive unilateral hydrocephalus at 5.5 months of age, immediately before ventriculoperitoneal shunt placement.

Figure 2 presents cranial ultrasound findings demonstrating unilateral periventricular hemorrhagic infarction (PVHI) in an extremely preterm newborn born at 23 + 2 gestational weeks (GWs), who subsequently developed unilateral hydrocephalus and underwent ventriculoperitoneal shunt placement at 6 months of age. Panels A–C demonstrate left-sided germinal matrix hemorrhage–intraventricular hemorrhage (GMH-IVH) with evolving PVHI on the 14th day of life (DOL). Panels D–F show left-sided hydrocephalus without signs of intracranial hypertension at 4 months of age. Panels G–H demonstrate hypertensive unilateral hydrocephalus at 5.5 months of age, immediately prior to ventriculoperitoneal shunt placement.

**Perinatal arterial ischemic stroke (PAIS):** PAIS results from occlusion of an arterial vessel and may frequently involve deep brain structures, particularly in the form of perforator strokes [55,59]. In a prospective hospital-based study of 73 newborn infants with perinatal arterial ischemic stroke (PAIS), 42% had a gestational age (GA) of ≤36 weeks. Among preterm infants, PAIS was left-sided in 61% and bilateral in 7%, with the middle cerebral artery (MCA) most frequently affected. Lenticulostriate branch involvement was common in infants with a GA of 28–32 weeks, whereas main MCA branch involvement was observed in almost all infants with a GA >33 weeks [55]. In a tertiary-care cohort study spanning 12 years, 55 patients were identified with a total of 79 perforator strokes; 45% of these patients were preterm [59].

**Cerebral sinovenous thrombosis (CSVT):** CSVT is rare, with a higher neonatal incidence of 2.6–2.69 per 100,000 newborns per year [60], compared to children. It is defined by thrombus formation within a cerebral venous structure, obstructing venous return and causing parenchymal injury of variable severity, from isolated intracranial hypertension to ischemia or hemorrhage. Clinical presentation may include seizures, lethargy, focal neurological deficits, or nonspecific symptoms, which can delay diagnosis [52,54]. Neonatal CSVT is multifactorial, usually reflecting genetic predisposition combined with acquired risk factors such as perinatal infection, dehydration, umbilical catheterization, and, rarely, lumbar puncture [52,54,60]. In a three-case series, one premature infant born at 34 gestational weeks developed CSVT in the setting of neonatal infection complicated by meningitis [60].

A retrospective consecutive cohort study included preterm infants with radiologically confirmed cerebral venous sinus thrombosis (CVST). MRI/MR venography (MRV) and computed tomography (CT)/CT venography (CV) scans were re-reviewed to define thrombus characteristics and brain injury patterns and to correlate them with the outcomes [61].

The cohort comprised 26 preterm infants: 65% moderate-to-late preterm, 27% very preterm, and 8% extremely preterm. Overall, 73% were symptomatic at presentation, most commonly with seizures or abnormal examination findings. Thrombosis primarily involved the transverse sinus (85%) and superior sagittal sinus (42%). Brain injury was mainly periventricular (35%) or deep white matter (31%), and intraventricular hemorrhage occurred in 46%. Anticoagulation was given to roughly 2/3 of the patients and was well tolerated. Outcomes were normal in 50%, mildly to moderately impaired in 25%, and severely impaired in 25% [61].

### 2.2. Risk Factors

The developmental characteristics of the preterm brain predispose this population to distinct mechanisms of stroke. Unlike term neonates, preterm infants may not present with overt seizures, which can delay recognition and diagnosis. Nonspecific clinical abnormalities, such as apnea, are common in this population and may be misinterpreted as manifestations of prematurity rather than indicators of cerebrovascular injury. Consequently, many cases, particularly perforator strokes, are identified incidentally on neuroimaging [59].

Current evidence suggests that strokes in preterm infants are usually multifactorial rather than attributable to a single causative factor. Recognized independent risk factors for PAIS include fetal cardiac rhythm abnormalities, neonatal hypoglycemia, twin-to-twin transfusion syndrome, dehydration, infection, patent ductus arteriosus, and prothrombotic disorders [55,57,62]. The coexistence of more than three potential risk factors, including maternal age, race, infertility, miscarriage, intrauterine growth restriction, and gestational diabetes, has been associated with a greater increase in stroke risk than any individual factor alone [62]. Limited studies have also frequently linked placental abnormalities, including placental weight below the 10th percentile, placental infarction, malperfusion, and chorioamnionitis, with PVHI [63]. Infection, prothrombotic disorders, and dehydration are likewise commonly reported risk factors for CSVT [54].

### 2.3. Clinical Manifestations

Perinatal stroke in preterm infants often presents with subtle and nonspecific clinical signs. Common manifestations include focal or generalized seizures, lethargy, hypotonia, irritability, feeding difficulties, and apnea. Because these findings are not specific to cerebrovascular injury, diagnosis may be delayed until later manifestations emerge, including hemiparesis, developmental delay, or early hand preference in the absence of recognized acute neonatal symptoms.

### 2.4. Diagnostic Evaluation

Neuroimaging is essential for the diagnosis of PVHI, particularly because clinical manifestations may be mild or easily overlooked. Cranial ultrasonography is generally preferred as the initial bedside modality for detecting these abnormalities, and multiple studies have confirmed its diagnostic accuracy and reliability [57]. Although cranial ultrasonography is effective for screening and serial monitoring, magnetic resonance imaging (MRI) provides more detailed anatomical characterization. MRI is superior for identifying associated white matter or cerebellar injury and supports more accurate prognostic assessment [57]. Serial CUS remains central to the diagnosis and follow-up of PVHI, whereas MRI with diffusion-weighted imaging (DWI) sequences is key for confirming PAIS, and MR venography (MRV) is indicated when CSVT is suspected [64,65]. Diffusion tensor imaging (DTI) enables evaluation of white matter microstructure within the posterior limb of the internal capsule and may assist in estimating the risk of subsequent motor impairment [66]. DTI-detected asymmetry has been shown to be a reliable predictor of unilateral spastic cerebral palsy, thereby serving mainly as a prognostic rather than a primary diagnostic tool [57,66].

### 2.5. Treatment

Management of perinatal stroke in preterm infants is primarily supportive and focuses on stabilization of vital parameters, pharmacological control of seizures, treatment of hydrocephalus when present, initiation of early rehabilitation therapies, and identification and management of underlying etiologic factors such as correction of dehydration and anemia and close monitoring and management of metabolic disturbances. Emerging therapeutic approaches, including stem cell-based interventions, are currently under investigation as potential strategies to improve neurodevelopmental outcomes and reduce long-term disability [54]. The use of anticoagulation for CSVT in neonates and in children with intracranial hemorrhage remains controversial, and existing pediatric professional consensus statements are not fully aligned on this issue. According to the joint consensus guideline of the European Pediatric Neurology Society (EPNS) and the French Society for Pediatric Neurology (SFNP), in the absence of contraindications, anticoagulation may be considered on an individual basis during the acute phase of neonatal CSVT for a duration of 6–12 weeks [67].

Repeated MRI performed at the end of this period may determine whether extension of treatment is needed [61].

### 2.6. Our Insight into Perinatal Stroke

Over a period exceeding 10 years, our department diagnosed fewer than a dozen cases of perinatal stroke among preterm infants, most of which were cases of periventricular hemorrhagic infarction (PVHI). A comprehensive review of these cases could provide a basis for more detailed investigation of the underlying pathophysiological mechanisms; however, such an analysis is beyond the scope of the present literature review.

During the 10-year period, we identified only one case of cerebral sinovenous thrombosis (CSVT) in a preterm newborn.

**Case report 1.** A male preterm neonate, born at 36 weeks’ gestation by spontaneous vaginal delivery, had a birth weight of 4030 g, length of 53 cm, and Apgar score of 6/6. The mother was a 41-year-old multipara, with an unremarkable personal and previous obstetric history; this pregnancy was monitored because of preeclampsia and polyhydramnios. On admission, the neonate was hypertrophic, Cushingoid in appearance, dyspneic, and pink-livid, with facial hematoma and hypotonia.

**Figure 3:**
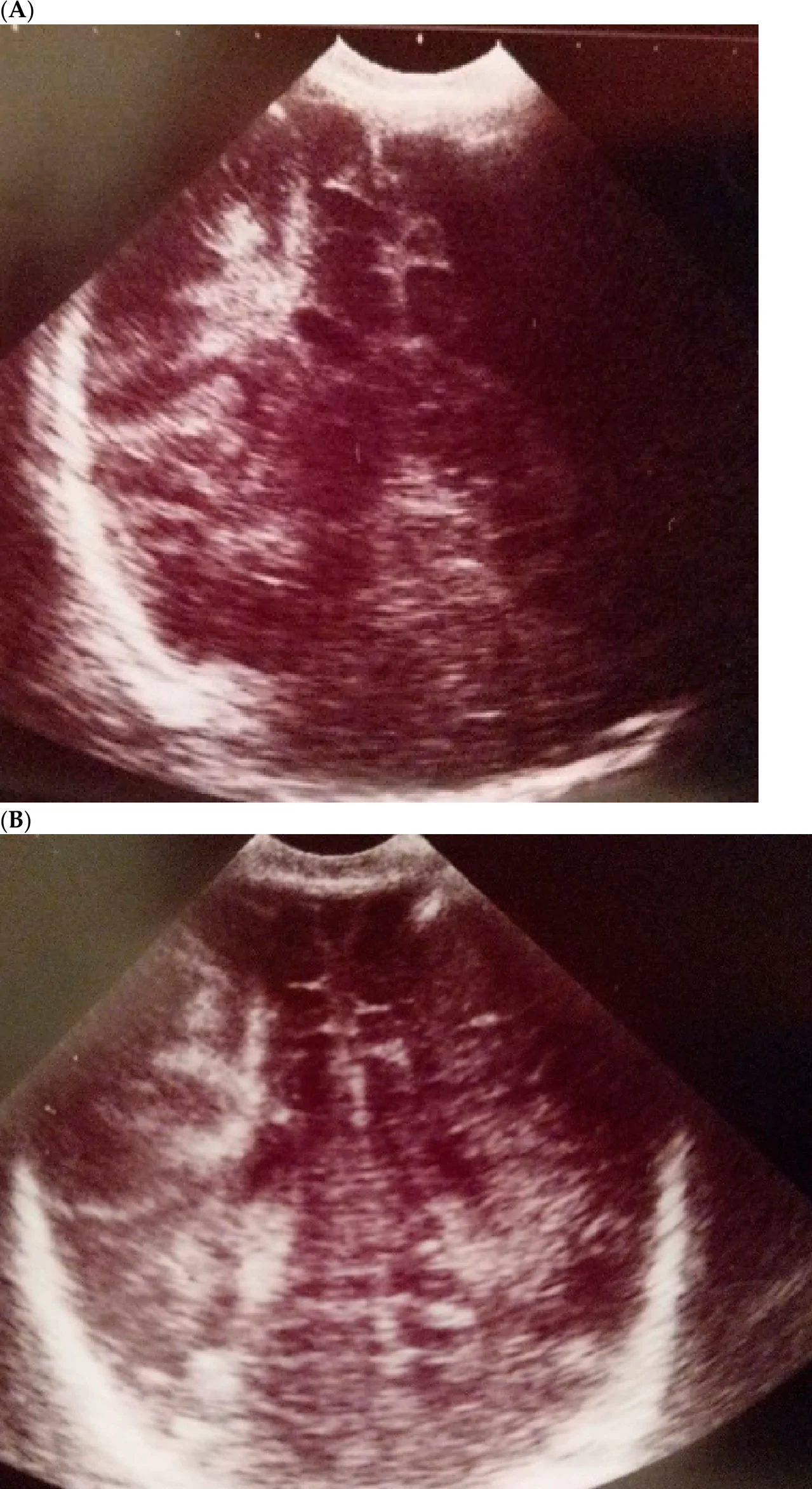

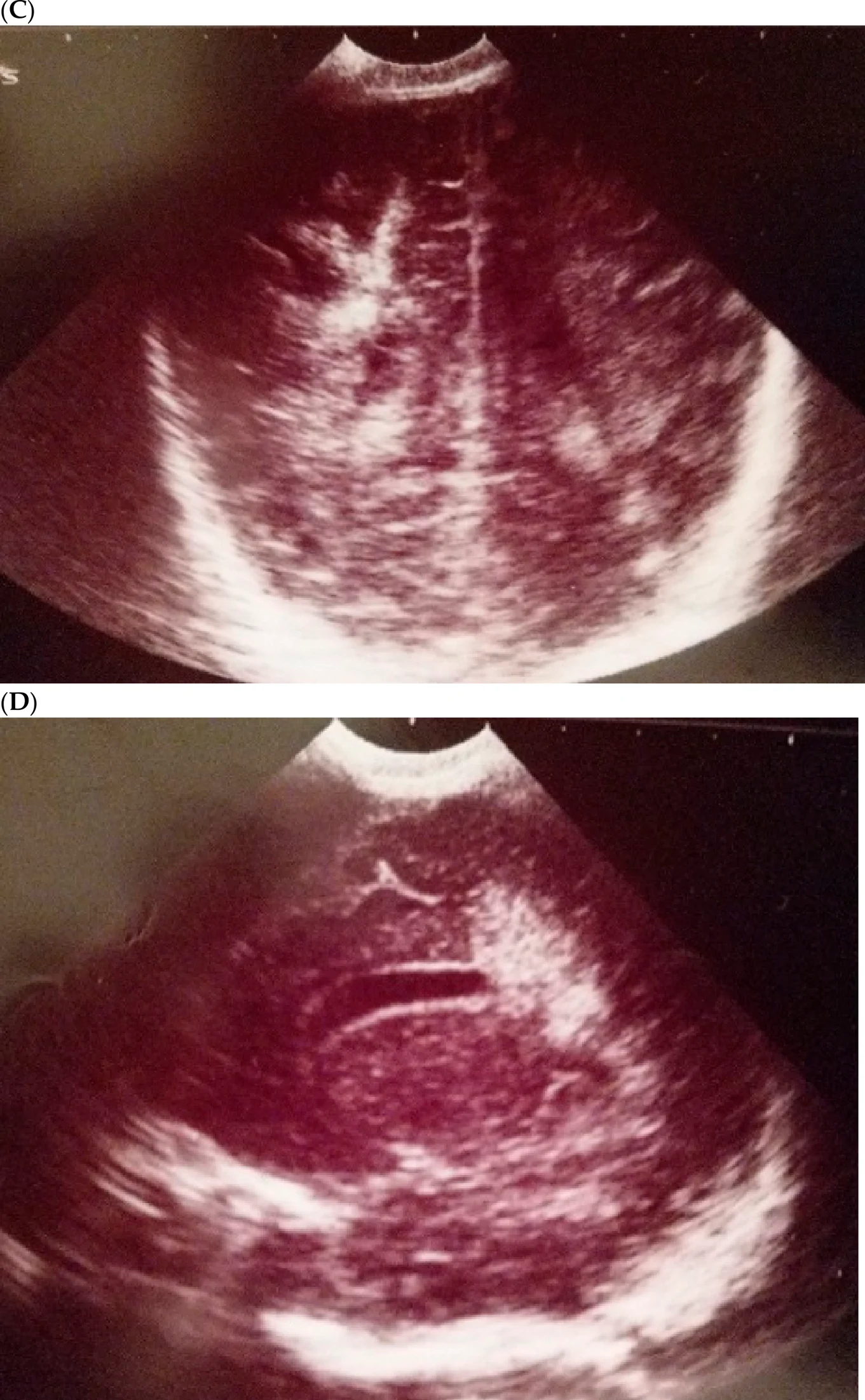
(**A**–**D**) Cranial ultrasound findings suggestive of a unilateral vascular event.

Diagnostic findings. Cerebrospinal fluid analysis indicated hemorrhage and hypoglycorrhachia. Fundoscopy revealed bilateral round retinal hemorrhages at the posterior pole. Echocardiography showed a moderately wide patent ductus arteriosus. Cranial ultrasound demonstrated dilated lateral ventricles with rounded echogenic margins and moderate-to-marked hyperechogenicity in the left III–V coronal sections and left parasagittal parieto-occipital region, with complete regression by discharge. Brain MRI on day 13 confirmed late subacute intracerebral hemorrhage and straight sinus thrombosis; follow-up MRI on day 28 showed near-complete regression (Figure 3).

Clinical course and outcome. Due to progressive postnatal dyspnea, the neonate was intubated, mechanically ventilated, and treated with intratracheal surfactant. After brief ventilation, he was extubated and remained on spontaneous breathing with incubator oxygen therapy until day 8. Empirical antibiotics, phenobarbital, and vitamin K were administered, with correction of hypoglycemia and hypocalcemia. Enteral feeding was introduced early and titrated. Transient apnea-associated subtle convulsions occurred on days 3 and 4 without recurrence. Indirect hyperbilirubinemia during the first week was managed conservatively. Cranial ultrasound suggested a vascular event, prompting MRI evaluation. Pre-discharge follow-up MRI was nearly normal. The neonate was discharged after 29 days of hospitalization, at 41 weeks’ corrected age, with normal somatic and neurological status.

Figure 3 depicts cranial ultrasound findings in panels A–D, suggesting a unilateral vascular event. Control MRI showed normal signal intensity in the straight sinus, resorption of blood from the occipital horns of the lateral ventricles, and small punctiform collections in the right temporal periventricular region, bilaterally in the occipital periventricular region, and in the posterior limb of the left internal capsule, without post-resorptive sequelae.

During this period of about a decade, we also identified a case of unspecified cerebral arterial stroke in a very preterm newborn delivered to a mother with autoimmune disease, with an unfavorable clinical outcome.

**Case report 2.** The patient was a female newborn from the mother’s second pregnancy, delivered by emergency cesarean section at 31 + 5 GW. During pregnancy, the mother was diagnosed with Sjögren’s syndrome and was treated with prednisolone and hydroxychloroquine. On the day preceding admission, she reported distinct fetal motor restlessness, followed by a sudden reduction in fetal movements.

At birth, the newborn’s parameters were as follows: birth weight of 1400 g, birth length of 38 cm, and Apgar scores of 1 and 3 at 1 and 5 min, respectively. The newborn was immediately intubated and mechanically ventilated. She had no spontaneous respiration, no spontaneous motor activity, generalized hypotonia, and no response to painful stimuli. The pupils were dilated and non-reactive. No external physical anomalies were observed.

**Figure 4:**
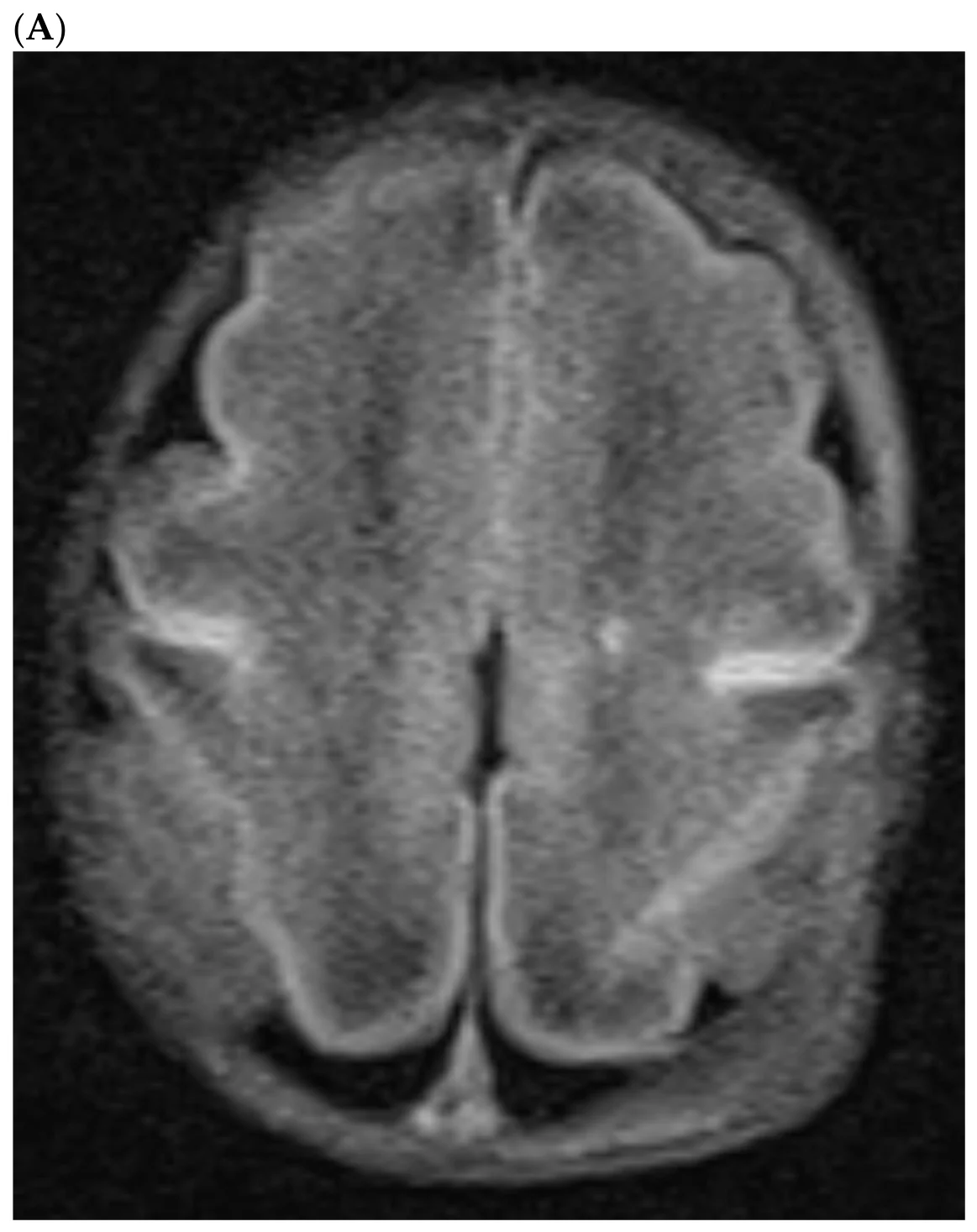

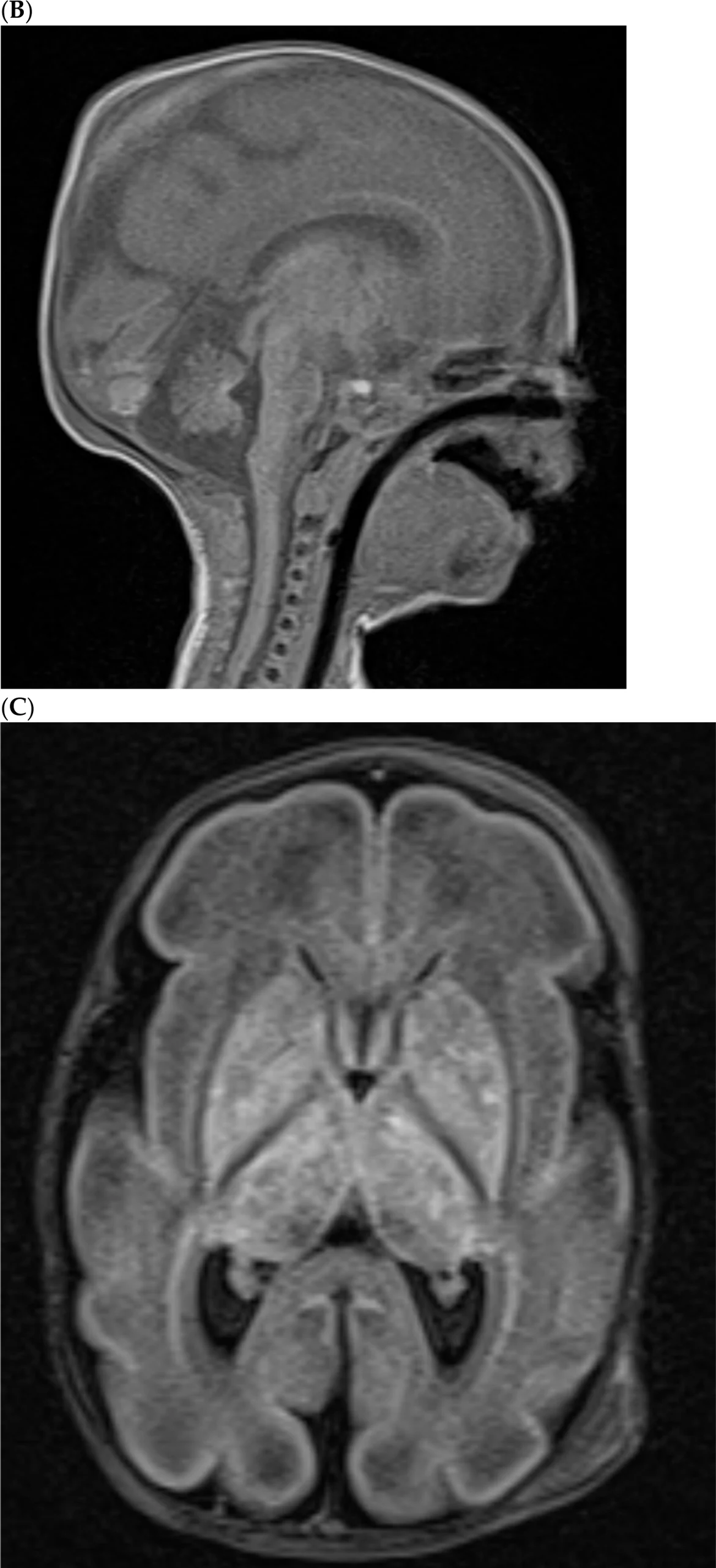

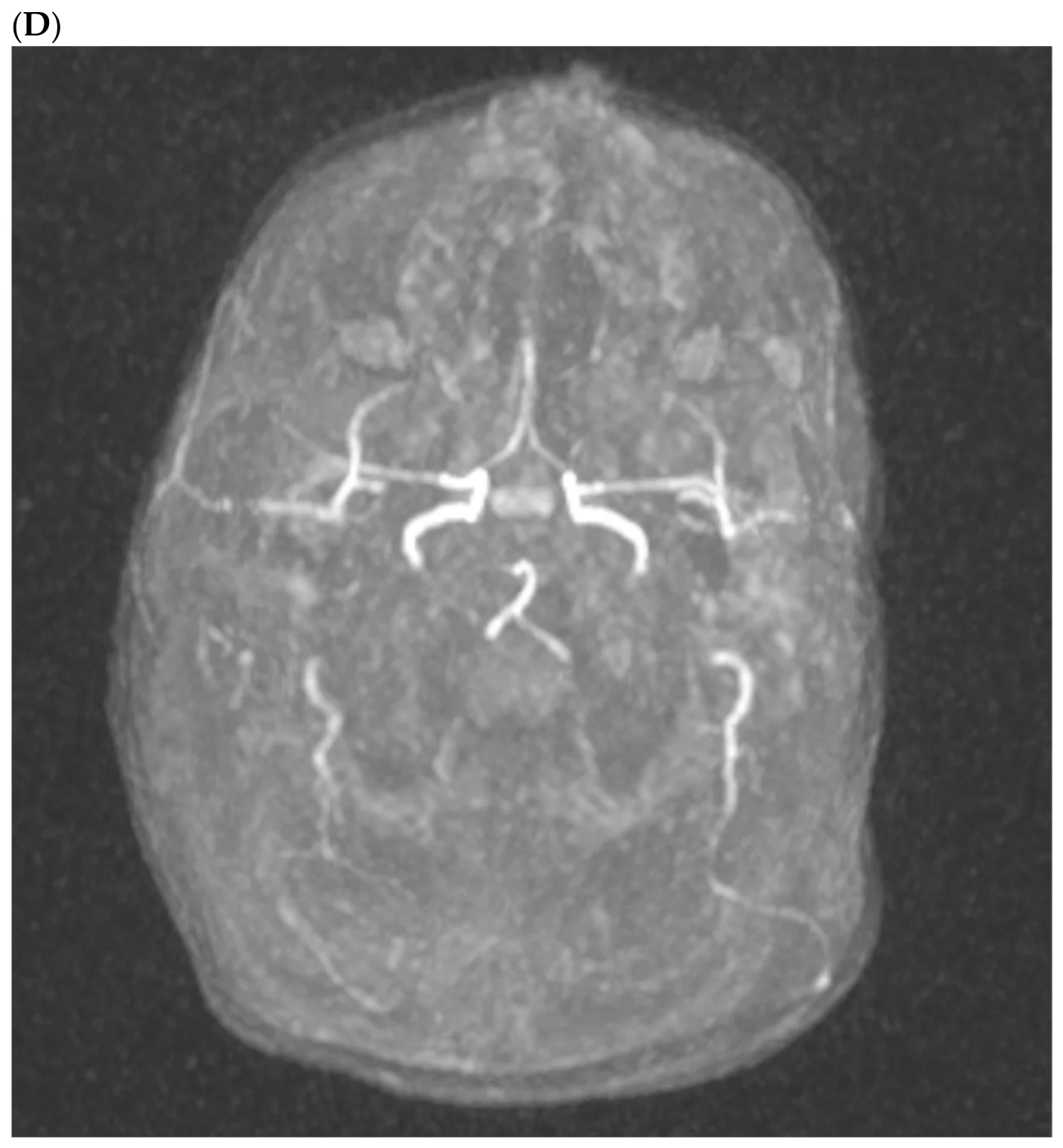
Magnetic resonance imaging (MRI) findings consistent with diffuse edema of the cerebral hemispheres. (**A**–**D**) Diffusely reduced corticomedullary differentiation on MRI, with extensive zones of diffusion restriction demonstrated on diffusion-weighted imaging/apparent diffusion coefficient (DWI/ADC) sequences.

Diagnostic findings. Echocardiography demonstrated a patent foramen ovale and a patent ductus arteriosus. Cranial ultrasound showed a markedly abnormal, “wiped-out” cerebral structure. Electroencephalography findings were consistent with severe hypoxic–ischemic encephalopathy. Brain magnetic resonance imaging demonstrated diffusely reduced corticomedullary differentiation, consistent with diffuse edema of the cerebral hemispheres. Extensive areas of diffusion restriction were identified on the diffusion-weighted imaging/apparent diffusion coefficient (DWI/ADC) sequences (Figure 4). Immune antibodies were detected in the newborn’s serum.

Histopathological examination of the placenta revealed hyper-torsion of the umbilical cord and evidence of compromised fetal perfusion.

Clinical course and outcome. Despite intensive care unit treatment, the newborn developed progressive multiorgan failure by the eighth day of life. The clinical course was fatal, and death occurred on the tenth day of life.

Figure 4A–D show diffusely reduced corticomedullary differentiation on the MRI consistent with diffuse edema of the cerebral hemispheres. Extensive zones of diffusion restriction are seen in the DWI/ADC sequences.

Case discussion. The initial clinical examination was consistent with neonatal coma. Diagnostic evaluation supported the diagnosis of a perinatal cerebrovascular insult. Potential contributing factors included congenital lupus syndrome related to maternal autoimmune disease, hyper-torsion of the umbilical cord, and placental findings suggestive of impaired fetal perfusion. A multifactorial etiology may also be considered.

### 2.7. Conclusions

The etiology of stroke in preterm infants remains incompletely elucidated, and the precise causal mechanisms require further investigation. Prognostic assessment is often complicated by comorbidities related to prematurity. Early detection depends on vigilant clinical observation and systematic identification of high-risk preterm infants. In high-risk preterm populations, increased awareness of clinical manifestations, combined with routine serial CUS and complemented by targeted MRI based on clinical suspicion and stroke subtype, may improve case ascertainment and warrant more accurate estimates of disease burden. Finally, PVHI represents the predominant stroke phenotype in preterm infants, whereas PAIS and CSVT, although less common, require distinct diagnostic and therapeutic approaches.

## Figures and Tables

**Table 1:** Mechanistic sequence of premature white matter injury: from upstream insults to oligodendrocyte dysmaturation.

Stage	Principal Mechanisms and Mediators	Pathophysiological Consequence
1. Predis posing vulnerability (third trimester)	Vascular end and border zones in cerebral white matter; impaired, frequently pressure-passive cerebrovascular autoregulation; white matter dominated by pre-OLs and densely populated by microglia	White matter is structurally and functionally primed for both ischemic and inflammatory injury
2. Upstream insults: hypoxia–ischemia and inflammation	Reduced CBF; maternal/fetal infection signaled by LPS and other PAMPs; inflammation sensitizes the immature brain to hypoxia–ischemia; vasoactive cytokines (TNF-α, IL-1β) further compromise cerebral perfusion	Insults may act independently or in concert, each amplifying the other
3. Glial activation	Microglia (activated via TLR4/CD14) and reactive astrocytes release IFN-γ, TNF-α, and IL-1β; generate superoxide and iNOS-derived NO; release glutamate while losing capacity to clear it	Local milieu becomes actively hostile to developing oligodendrocytes
4. Oxidative, excitotoxic, and mitochondrial injury	Energy failure reverses glutamate transporters, raising extracellular glutamate → activation of Ca^2+^-permeable (GluR2-deficient) AMPA/kainate and NMDA receptors on pre-OLs → Ca^2+^ influx → ROS/RNS and ONOO^−^ formation; immature antioxidant defenses and iron acquired for differentiation favor hydroxyl radical formation; NO acts as a mitochondrial poison, triggering AIF translocation	Excitotoxicity and free radical attack reinforce one another
5. Pre-oligodendrocyte damage	AMPA/kainate receptor activation at the cell soma; NMDA receptor activation on cell processes; NO-mediated mitochondrial (AIF) pathway	Apoptotic and, via NO, caspase-independent pre-OL death; loss of cell processes; failed axonal ensheathment, compromising the earliest stage of myelination
6. Impaired maturation (dysmaturation)	Progenitors proliferate and replenish the pre-OL pool, but persistent microglial and astroglial activation and ongoing inflammation block their differentiation into mature myelinating oligodendrocytes	Maturation arrest, rather than cell loss alone, results in diffuse hypomyelination, axonal and secondary neuronal disturbances, and long-term neurodevelopmental impairment

Abbreviations: AIF, apoptosis-inducing factor; AMPA, α-amino-3-hydroxy-5-methyl-4-isoxazolepropionic acid; CBF, cerebral blood flow; CD14, cluster of differentiation 14; GluR2, glutamate receptor subunit 2; IFN-γ, interferon-γ; IL-1β, interleukin-1β; iNOS, inducible nitric oxide synthase; LPS, lipopolysaccharide; NMDA, N-methyl-D-aspartate; NO, nitric oxide; ONOO^−^, peroxynitrite; PAMPs, pathogen-associated molecular patterns; pre-OL, premyelinating oligodendrocyte; RNS, reactive nitrogen species; ROS, reactive oxygen species; TLR4, Toll-like receptor 4; TNF-α, tumor necrosis factor-α.

**Table 2:** Influence of chorioamnionitis on brain injury and neurodevelopmental outcome in preterm infants: key clinical studies.

Reference	Design/n/Outcome	Crucial Finding
Wu YW, Colford JM Jr. JAMA, 2000. [17]	*Meta-analysis* (random effects; case–control and cohort studies) *n* = 19 studies (clinical chorioamnionitis); *n* = 7 studies (HCA) Outcome: CP and cPVL in preterm and term infants	In preterm infants, clinical chorioamnionitis was associated with CP (RR 1.9; 95% CI 1.4–2.5) and cPVL (RR 3.0; 95% CI 2.2–4.0). HCA was associated with cPVL (RR 2.1; 95% CI 1.5–2.9) but not significantly with CP (RR 1.6; 95% CI 0.9–2.7). In term infants, clinical chorioamnionitis was associated with CP (RR 4.7; 95% CI 1.3–16.2). Differences between studies were explained by inconsistent definitions of clinical chorioamnionitis, the extent of blinding, and adjustment for confounders.
Anblagan D et al. Sci Rep., 2016. [18]	*Cohort* with placental histology *n* = 90 preterm infants; HCA *n* = 26 (29%) Outcome: MRI at TEA (TBSS of FA; volumetry of whole brain, tissue compartments, and cerebrospinal fluid)	HCA was associated with decreased FA in the genu, cingulum cingulate gyri, centrum semiovale, inferior longitudinal fasciculi, limbs of the internal capsule, external capsule, and cerebellum (*p* < 0.05, corrected). The association was independent of the degree of prematurity, BPD, and postnatal sepsis, suggesting that diffuse WMI begins in utero in a significant proportion of preterm infants.
Bierstone D et al. JAMA Pediatr., 2018. [19]	*Prospective multicenter cohort* (3 academic centers: Canada, the Netherlands, United States) *n* = 350, 24–32 weeks’ GA; HCA *n* = 145 (41.4%) Outcome: early MRI (IVH, punctate WMI); Bayley-III at 18–24 months’ corrected age	HCA was associated with lower GA (median 26.4 vs. 28.0 weeks) but not with IVH, punctate WMI, or motor outcome (adjusted Bayley-III motor score −2.2; 95% CI −5.6 to 1.3). Cognitive scores were marginally lower in univariable analysis, but the difference was attenuated after adjustment (−3.0; 95% CI −6.4 to 0.4). Postnatal factors, such as infection, attenuated the association.
Jain VG et al. Am J Obstet Gynecol., 2022. [20]	*Prospective multicenter cohort* (CINEPS) *n* = 353, ≤32 weeks’ GA; moderate-to-severe acute HCA *n* = 56 vs. no or mild HCA *n* = 297 Outcome: 3-T MRI at TEA (brain abnormality score, total brain volume, cortical maturation)	Moderate-to-severe acute HCA was associated with a higher brain abnormality score (median 7 vs. 4; *p* < 0.001; adjusted β = 2.84 [1.51–4.16]), lower total brain volume (*p* = 0.03), and reduced sulcal depth (*p* = 0.02). In mediation analysis, 50% of the brain abnormalities were an indirect consequence of preterm birth and 50% a direct effect of HCA. Funisitis was associated with the brain abnormality score (*p* = 0.005) but had no significant direct effect once the indirect effect of preterm birth was accounted for.
Peterson LS et al.; CINEPS Investigators. JAMA Netw Open, 2025. [21]	*Prospective population-based cohort* (CINEPS; 5 hospitals in southwestern Ohio) *n* = 304, ≤32 weeks’ GA Outcome: Bayley-III motor (primary), cognitive, and language scores at 22–26 months’ corrected age	Moderate-to-severe HCA was associated with lower motor (β −7.0; 95% CI −11.2 to −2.8), cognitive (β −6.0; 95% CI −10.2 to −1.8), and language scores (β −8.8; 95% CI −14.5 to −3.2). Exposed infants were born a median of 2.6 weeks earlier. Earlier birth mediated 25% of the association with motor scores (β −1.7; 95% CI −2.9 to −0.6), whereas 75% was a direct association of HCA or inflammation (β −5.3; 95% CI −9.9 to −0.7).

Abbreviations: Bayley-III, Bayley Scales of Infant and Toddler Development, Third Edition; BPD, bronchopulmonary dysplasia; CI, confidence interval; CINEPS, Cincinnati Infant Neurodevelopment Early Prediction Study; CP, cerebral palsy; cPVL, cystic periventricular leukomalacia; FA, fractional anisotropy; GA, gestational age; HCA, histologic chorioamnionitis; IVH, intraventricular hemorrhage; MRI, magnetic resonance imaging; RR, relative risk; TBSS, tract-based spatial statistics; TEA, term-equivalent age; WMI, white matter injury.

## Data Availability

No publicly available datasets were generated or analyzed in the preparation of this manuscript. The article is based primarily on a review of previously published literature, and the limited retrospective clinical material described in the manuscript consists of anonymized cases from the authors’ institutional teaching and publication archive. Owing to ethical and privacy restrictions, individual-level clinical data and original imaging files are not publicly available. Further information may be made available by the corresponding author upon reasonable request and subject to applicable institutional and ethical approvals.

## Abbreviations

The following abbreviations are used in this manuscript:

ADC	Apparent diffusion coefficient
AIF	Apoptosis-inducing factor
AIS	Arterial ischemic stroke
AMPA	α-Amino-3-hydroxy-5-methyl-4-isoxazolepropionic acid
APPIS	Arterial presumed perinatal ischemic stroke
aRR	Average risk ratio
Bayley-III	Bayley Scales of Infant and Toddler Development, Third Edition
BPD	Bronchopulmonary dysplasia
CAP	Caffeine for Apnea of Prematurity (trial)
CBF	Cerebral blood flow
CD14	Cluster of differentiation 14
CI	Confidence interval
CINEPS	Cincinnati Infant Neurodevelopment Early Prediction Study
CP	Cerebral palsy
cPVL	Cystic periventricular leukomalacia
CSVT	Cerebral sinovenous thrombosis
CT	Computed tomography
CUS	Cranial ultrasonography
CV	Computed tomography venography
CVST	Cerebral venous sinus thrombosis
DEHSI	Diffuse and excessive high signal intensity
DOL	Day of life
DTI	Diffusion tensor imaging
DWI	Diffusion-weighted imaging
EEG	Electroencephalography
EPO	Erythropoietin
FA	Fractional anisotropy
GA	Gestational age
GluR2	Glutamate receptor subunit 2
GMH-IVH	Germinal matrix hemorrhage–intraventricular hemorrhage
GW	Gestational weeks
HCA	Histologic chorioamnionitis
HIE	Hypoxic–ischemic encephalopathy
HS	Hemorrhagic stroke
IFN-γ	Interferon-γ
IL-1β	Interleukin-1β
iNOS	Inducible nitric oxide synthase
IVH	Intraventricular hemorrhage
LPS	Lipopolysaccharide
MCA	Middle cerebral artery
MRI	Magnetic resonance imaging
MRV	Magnetic resonance venography
MSC-Exos	Mesenchymal stem cell-derived exosomes
MSCs	Mesenchymal stem cells
NEC	Necrotizing enterocolitis
NMDA	N-methyl-D-aspartate
NO	Nitric oxide
ONOO^−^	Peroxynitrite
PAIS	Perinatal arterial ischemic stroke
PAMPs	Pathogen-associated molecular patterns
PENUT	Preterm Erythropoietin Neuroprotection Trial
PPHS	Presumed perinatal hemorrhagic stroke
PPROM	Preterm premature rupture of membranes
pre-OL	Premyelinating oligodendrocyte
PVHI	Periventricular hemorrhagic infarction
PVin	In utero periventricular venous infarction
PVL	Periventricular leukomalacia
PVWMLs	Punctate white matter lesions
RNS	Reactive nitrogen species
ROS	Reactive oxygen species
RR	Relative risk
SNP	Single-nucleotide polymorphism
TBSS	Tract-based spatial statistics
TEA	Term-equivalent age
TH	Therapeutic hypothermia
TLR4	Toll-like receptor 4
TNF-α	Tumor necrosis factor-α
WMI	White matter injury
